# Benchmarking Machine Learning Models for HIV‑1 Protease Inhibitor Resistance Prediction: Impact of Data Set Construction and Feature Representation

**DOI:** 10.1021/acs.jcim.5c01544

**Published:** 2025-09-25

**Authors:** Rocío Lucía Beatriz Riveros Maidana, Lucas de Almeida Machado, Ana Carolina Ramos Guimarães

**Affiliations:** † Laboratório de Genómica Aplicada e Bioinovac̨ões, 196605 Instituto Oswaldo Cruz/Fiocruz, Rio de Janeiro 21040-900, Brazil; ‡ Programa de Pós-Graduac̨ão em Biologia Computacional e Sistemas, Instituto Oswaldo Cruz/Fiocruz, Rio de Janeiro 21040-900, Brazil; § Institute of Technology on Immunobiologicals (Bio-Manguinhos) - Fiocruz, Rio de Janeiro 21040-900, Brazil

## Abstract

The rapid emergence of drug resistance in viral infections represents a significant global health challenge, threatening the efficacy of treatments for multiple diseases. Machine learning models have emerged as valuable tools for predicting antiviral drug resistance from genomic data, with HIV-1 protease serving as a well-characterized model system due to its extensive experimental data and clinical relevance. Here, we systematically evaluate multiple previously published HIV-1 protease inhibitor (PI) resistance prediction models across three distinct data sets with different preprocessing and ambiguous sequencing processing strategies and propose a new approach for preprocessing. We tested Steiner’s data set (*n* = 1540) with first-amino-acid selection at ambiguous positions, Shen’s expanded data set (*n* = 500,390) with all possible combinations at ambiguous positions, and our In-house data set (*n* = 869) with strict exclusion of ambiguous sequences. We compare neural networks architectures (Multilayer Perceptron, Bidirectional Recurrent Neural Network, and Convolutional Neural Network), traditional machine learning models (Random Forest and K-Nearest Neighbor), and logistic regression using either zScales physicochemical descriptors or Rosetta energy terms. Sequence expansion preprocessing can artificially increase performance metrics (mean AUC: 0.986–0.999) by creating substantial redundancy (99.6% of expanded data set consists of duplicated sequences from 2096 unique originals), while our clustering-based validation approach provides a more stringent assessment of model generalizability. Remarkably, our physicochemically informed logistic regression models achieved performance comparable to complex neural networks on challenging test sets (zScales LR: AUC = 0.973; Rosetta LR: AUC = 0.944), while offering superior interpretability. Furthermore, the zScales LR model offered significantly greater computational efficiency (0.007 s/prediction) compared to that of Rosetta LR (776.117 s/prediction). Mutual information analysis revealed distinct complementary resistance mechanisms: The zScales descriptors identified discrete resistance hotspots at positions 10, 46, 54, 71, and 90, while the Rosetta energy terms revealed interconnected energetic networks across structurally adjacent residues, particularly in functionally critical flap regions (positions 46–54). This study demonstrates how data set construction choices directly impact apparent model performance while establishing that well-chosen physicochemical feature representations can match or exceed complex neural networks for HIV-1 PI resistance modeling, offering both accuracy and mechanistic interpretability critical for clinical implementation and drug development.

## Graphical abstract

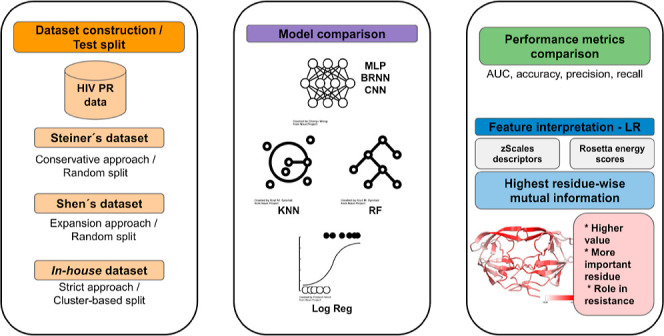

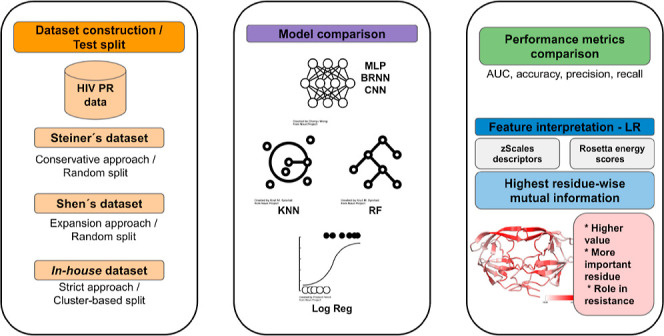

## Introduction

Drug resistance in viral infections is a major global health concern, reducing treatment efficacy and increasing disease burden.^1–4^ HIV-1 is a key model for studying this phenomenon due to its high mutation rate (∼10^–4^ per nucleotide per cycle) and abundant clinical and genotype–phenotype correlation data.^2,5^ The HIV-1 protease (PR), a homodimeric aspartyl enzyme with 99 residues per monomer, is essential for viral maturation through the cleavage of Gag and Gag-Pol precursors. As a critical target of antiretroviral therapy, several protease inhibitors (PIs) have been developed.^6^ However, the virus’s rapid evolution often leads to resistant variants, which points to the need for robust predictive tools to guide treatment.

## Evolution of HIV-1 Drug Resistance Prediction Approaches

Computational methods for predicting HIV-1 drug resistance from genetic sequences have advanced significantly over the past two decades. Early tools such as HIVdb^7^ and WebPSSM^8^ relied on expert-curated rules and position-specific scoring matrices, respectively. With the rise of machine learning, diverse models, such as random forests (e.g., SHIVA^9^), support vector machines (geno2pheno^10^), decision trees,^11^ logistic regression,^12^ and artificial neural networks,^13,14^ have been employed for resistance modeling. However, many sequence-based models fail to account for structural consequences of mutations that drive resistance.^15^

Traditional machine learning models remain effective, particularly in data-limited contexts. Random forests are widely used due to their robustness in high-dimensional, small-sample settings.^16,17^ Riemenschneider et al.^18^ showed that multilabel random forests leveraging cross-resistance information improve accuracy over single-drug models. Logistic regression, though simple, has proven useful when coupled with appropriate feature design.^19,20^ Its interpretability is a distinct advantage in clinical contexts, and recent studies^21,22^ show that it can perform on par with more complex models in small medical data sets, emphasizing the need to align model complexity with data availability.

Beyond sequence-based methods, structural and molecular approaches have been developed to incorporate 3D information into resistance prediction models. Wang et al.^15^ introduced a molecular field-based model using partial least-squares regression to generate 3D coefficient maps that highlight protease regions influencing drug binding. Similarly, Ota et al.^23^ proposed a molecular field mapping technique based on a 3D protein structure. Whitfield et al.^24^ integrated molecular dynamics simulations with machine learning to explore protein–ligand interactions, offering dynamic insights often missed by sequence data alone.

More recently, deep learning models have been applied to resistance prediction. Steiner et al.^25^ compared multilayer perceptrons (MLPs), bidirectional recurrent neural networks (BRNNs), and convolutional neural networks (CNNs), with CNNs showing superior performance, likely due to their ability to detect local sequence patterns. However, the effectiveness of such models is often constrained by limited data set sizes.^26^ Recent work in the broader field of machine learning for small medical data sets has emphasized the importance of careful model selection and validation strategies when working with limited data.^21,22^

## Data Processing and Feature Representation

Model performance also depends heavily on data preprocessing and feature representation. A critical challenge lies in handling sequence ambiguities, which occur when multiple viral variants coexist in a patient or due to limitations of sequencing methods.^23,27^ This genetic heterogeneity introduces ambiguities in genotype–phenotype correlations and presents technical challenges for many machine learning methods not designed to handle multiallelic codes directly.^27^ Several strategies have been developed to address this challenge, each with its distinct advantages and limitations. Sequence expansion into all possible variants^28^ maximizes data utilization and increases data set size while introducing redundancy and artificial sequences that (likely) do not exist. Others exclude sequences with ambiguities at drug resistance mutation (DRM) positions,^25^ which reduces data set diversity and depends on prior assumptions about DRMs. Some methods consist of selecting a single representative amino acid,^25^ a compromise that maintains data set size but may introduce bias if the selected amino acid is not representative of the dominant viral variant. Probabilistic methods have also been developed to handle incomplete sequencing data by weighting possible combinations based on their likelihood of occurrence.^23^

Another crucial factor is the method used to split data into training and test sets, as this directly impacts model evaluation and assessment of generalizability.^20^ Various procedures have been employed in studies predicting drug resistance. Some involve simple random splits,^29^ while others create test sets chronologically, reserving newer sequences for testing, mimicking real-world scenarios.^30^ Stratified cross-validation has been used to ensure each partition maintains the same proportion of resistant to nonresistant sequences.^25^

Feature representation constitutes another critical aspect, significantly influencing both the model performance and interpretability. Traditional methods include binary encoding of mutations,^31^ which produces sparse high-dimensional vectors indicating the presence or absence of specific amino acid substitutions, and integer encoding,^25^ which assigns numerical values to each amino acid position, preserving sequence order for neural network models. Other methods aim to capture structural or biochemical information. For instance, Delaunay triangulation of Cα atoms^28^ has been used to extract a subset of interactions from protein structure in a compact 210-dimensional vector. K-mers and multi-*n*-grams^32^ capture local sequence patterns by representing short, overlapping sequences of amino acids. Physicochemical descriptors, such as zScales,^33^ use principal components of physicochemical properties like hydrophobicity and charge to represent amino acids in a continuous five-dimensional space, capturing the biochemical effects of mutations. Reduced amino acid alphabets^34^ simplify the input space by grouping residues with similar properties, preserving functional relevance while reducing dimensionality. Structural features derived from protein models^24^ integrate three-dimensional information about residue interactions and spatial relationships. Rosetta energy terms^35^ quantify the energetic consequences of mutations using physics- and knowledge-based potentials, having shown utility even in predicting amyloidogenicity.^29^ These representations enhance the predictive performance and facilitate model interpretability, which is essential for understanding the biological relevance of resistance mechanisms. Recently, Protein Language Model protein representations emerged as a new form of high-dimensional learned feature that can be used in classification tasks.^36^

## Validation Strategies

Validation strategies for resistance prediction models must address challenges specific to viral sequence data, including limited sample sizes, class imbalance, and distributional shifts between training and test data. Traditional random splitting approaches may overestimate model performance due to sequence similarity or shared resistance patterns among related viral isolates.^25,29,37^ More robust strategies include stratified cross-validation,^25^ addressing class imbalance issues common in resistance data, and time-based splitting, where more recent sequences are reserved for testing, mimicking real-world scenarios where models must predict resistance in newly emerging variants.^30^ Recent studies^38^ highlight the importance of validation adapted to small data sets. Hazbeh et al.^39^ demonstrated that preprocessing strategies such as outlier removal and feature selection can substantially enhance model performance, often rivaling the impact of model choice itself.

## Challenges in HIV-1 Resistance Prediction Models

Despite significant advances in HIV-1 drug resistance modeling, limited data availability for newer drugs and substantial class imbalance constrain model development and evaluation. These limitations impact both clinical utility and biological interpretability. Limited data availability for newer drugs, particularly second-line PIs, constrains model training and evaluation, as these medications often have fewer matched genotype–phenotype data points available.^25,38^ This data scarcity poses a particular challenge for deep learning architectures, which typically require large training sets to perform optimally.^26^ Another significant challenge is class imbalance, as resistant sequences usually represent the minority class, especially for newer drugs with higher genetic barriers to resistance.^25,37^ This imbalance can bias models toward the majority class, reducing sensitivity for detecting resistance and potentially leading to suboptimal treatment decisions. Finding models that are both accurate and interpretable remains a challenge, as highly accurate black-box models often lack biological interpretability.^40^ Ensuring computational efficiency for clinical implementation represents another practical challenge, particularly for structure-based methods that require intense calculations for feature generation. Importantly, as HIV represents one of the best-characterized systems for studying viral drug resistance mechanisms, the methodological advances and challenges identified here have broader implications for other viral pathogens. These challenges indicate the need for innovative frameworks that combine the strengths of different modeling strategies, such as hybrid models integrating sequence-based and structure-based features, ensemble methods leveraging multiple algorithms, and interpretable models providing mechanistic insights while addressing the specific complexities of HIV resistance data.

In this study, we benchmark HIV-1 PI resistance prediction models across three distinct data sets that differ in their preprocessing and validation strategies. While prior works have introduced various models, our contribution lies in systematically analyzing how data set construction impacts both predictive performance and biological interpretability. To this end, we compare a representative range of models, from traditional machine learning (RF and K-Nearest Neighbor)^28^ and established neural networks architectures (MLP, BRNN, and CNN),^25^ to two novel, interpretable logistic regression models based on physicochemical (zScales descriptors^41^) and structural (Rosetta energy terms^35^) representations. These models were selected to capture a spectrum of algorithmic complexity and feature types, ranging from raw sequences to structural and physicochemical descriptors, allowing us to evaluate the trade-off between interpretability and predictive power, particularly under data limitations and class imbalance. Unlike previous studies that focused on single data sets or model types, our work jointly evaluates model generalizability across data sets built with different modeling approaches, including one generated by us using a clustering-based validation strategy designed to produce biologically meaningful partitions. Our findings reveal that interpretable models based on structural and physicochemical features can match or surpass more complex models under certain conditions, providing both robust predictions and mechanistic insight. This integrated evaluation framework offers new perspectives for model selection in resistance modeling and supports the development of clinically relevant and interpretable tools for drug design and treatment planning.

## Methods

### Data

Genotype–phenotype data were obtained from the Stanford University HIV Drug Resistance Database^42^ for eight PIs: darunavir (DRV), fosamprenavir (FPV), atazanavir (ATV), indinavir (IDV), lopinavir (LPV), nelfinavir (NFV), saquinavir (SQV), and tipranavir (TPV) (downloaded 02/07/2023). We selected subtype B sequences with corresponding PhenoSense assay,^43^ which measures the levels of resistance to a PI compared to the wild-type sequence by determining the concentration of drug that inhibits viral replication in tissue culture.^44^ Sequences were classified as resistant or susceptible based on established clinical cutoffs of fold change in IC50 for PIs,^19^ which are consistent with those used by the Stanford HIV Drug Resistance Database, 2.0 for TPV, 3.0 for NFV/SQV/IDV/ATV, 4.0 for FPV, 9.0 for LPV, and 10.0 for DRV.^45,46^ If the fold change value is less than the cutoff value, the mutant is classified as nonresistant or susceptible to the drug and reported as 0. Otherwise, the mutant is considered as resistant and reported as 1.

### Data Set Construction and Preprocessing Approaches

We processed the data to create three data sets using different preprocessing strategies, each addressing specific challenges in HIV sequence data analysis.

For our In-house data set, the sequences were filtered with custom R scripts (R version 3.6.1) to include only those containing exactly 99 amino acids. Sequences with insertions, deletions, stop codons, or multiple mutations at the same sites were excluded. Each set of redundant sequences (defined as sequences with 100% amino acid identity) was represented by a single representative sequence to which was attributed the Phenosense resistance assay value equal to the median of all the identical sequences it represents. By retaining median resistance values for identical sequences, we addressed potential inconsistencies in experimental measurements while maintaining a diverse representation of the sequence variants.

The Shen data set^28^ started from the same Stanford HIV Drug Resistance Database and applied identical initial filtering criteria: selection of subtype B sequences with exactly 99 amino acids. Subsequently, the data set employed sequence expansion at ambiguous positions using Python v3.10.8, where sequences containing ambiguous amino acids were expanded into multiple sequences sharing identical resistance values. For example, a sequence with an ambiguity at one position encoding two possible amino acids would generate two distinct sequences in the final data set, both sharing the same resistance value. After expansion, sequences with insertions, deletions, or stop codons were excluded.

The Steiner data set^25,47^ was derived from the same Stanford HIV Drug Resistance Database but employed a different preprocessing protocol. They excluded redundant sequences from intrapatient data and sequences with ambiguities at major DRM positions. For the remaining sequences with ambiguities, they selected the first listed amino acid at each position and excluded incomplete sequences.

### Test Data Set Construction

For model evaluation, we employed diverse strategies for test set construction, emphasizing a novel clustering-based approach for the In-house data set. Our method employed a clustering-based strategy to ensure comprehensive representation of sequence diversity while providing a more stringent assessment of model generalizability compared to random splitting methods. Unlike random splits that may inadvertently place highly similar sequences in both training and test sets, our cluster-based sampling minimizes sequence similarity between train/test partitions by ensuring that sequences from each cluster are represented in both sets proportionally, allowing a better test model generalization by requiring predictions on sequences that are more evolutionarily distant from the training data. To do so, pairwise sequence alignments were performed using dynamic programming with the BLOSUM80 substitution matrix,^48^ followed by t-Distributed Stochastic Neighbor Embedding (*t*-SNE)^49^ dimensionality reduction using scikit-learn v1.5.2. *T-SNE* parameters were optimized iteratively by testing perplexity values from 5 to 50, each coupled with K-means clustering (*K* = 2–10). The optimal configuration was selected based on the highest silhouette score across all data points, which indicates the best balance between intracluster cohesion and intercluster separation. The test set (20%) was constructed by sampling equally from each cluster with the remaining 80% used for training with 5-fold stratified cross-validation. Both Shen’s^28^ and Steiner’s^42^ data sets used random 80:20 splits implemented with scikit-learn v1.5.2.

### Machine Learning Models

To benchmark how different test data set construction strategies impact model performance evaluation, we trained seven machine learning models: three replicating Steiner^25^—MLP, BRNN, and CNN; two replicating Shen et al.^28^—RF and K-Nearest Neighbor; and two novel Logistic Regression models developed in-house. All models were trained using 5-fold stratified cross-validation on the training data sets. Subsequently, we evaluated all models on the three test data sets resulting from different construction strategies (Shen’s, Steiner’s, and our In-house approach) to systematically assess how test set composition influences performance metrics. The model comparison aimed to evaluate performance across different data set types and assess the value of physicochemically informed feature representations. To ensure comparability with prior work and isolate the effects of data set design, we retained the original hyperparameters and model architectures defined in the previous studies.

Table 1 summarizes the control parameters used. For published models (MLP, BRNN, CNN, KNN, and RF), we preserved the original specifications; for our models (zScales LR and Rosetta LR), we applied standardized hyperparameter selection.

**Table 1:** Control Parameters for Each Machine Learning Algorithm Used in This Study

model	feature representation	architecture/algorithm parameters	regularization parameters	training parameters	implementation	reference
MLP	integer-encoded sequence	4 hidden layers (33 units each), ReLU activation, sigmoid output	L2 regularization	RMSprop optimizer, batch size = 64, epochs = 500, class weights = inverted class ratio (weight_0 = 1, weight_1 = n_susceptible/n_resistant)	R v3.6.1, Keras v2.11.1, TensorFlow v2.12.0	Steiner et al.[Bibr ref25] (2020)
BRNN	integer-encoded sequence	1 bidirectional LSTM layer (33 units), sigmoid output	dropout = 0.2, recurrent dropout = 0.2		R v3.6.1, Keras v2.11.1, TensorFlow v2.12.0	Steiner et al.[Bibr ref25] (2020)
CNN	integer-encoded sequence	2 convolutional layers (32 filters), Kernel size = 9, ReLU activation, 1 max pooling layer, Sigmoid output	N/A		R v3.6.1, Keras v2.11.1, TensorFlow v2.12.0	Steiner et al.[Bibr ref25] (2020)
KNN	Delaunay triangulation (210-dimensional vector)	K = 6	N/A	N/A	Python v3.10.8, scikit-learn v1.5.2	Shen et al.[Bibr ref28] (2016)
RF	Delaunay triangulation (210-dimensional vector)	number of trees = 10	N/A		Python v3.10.8, scikit-learn v1.5.2	Shen et al.[Bibr ref28] (2016)
zScales LR^a^	zScales physicochemical properties(495-dimensional vector)	logistic classifier	L1 regularization, C = {0.01, 0.1, 1, 10, 100} (optimized via 5-fold CV using accuracy)	class weights = inverted class ratio	Python v3.10.8, scikit-learn v1.5.2	Novel (this study)
Rosetta LR^a^	REF15 energy terms (1882-dimensional vector)	logistic classifier		class weights = inverted class ratio	Python v3.10.8, scikit-learn v1.5.2	Novel (this study)

a For both zScales LR and Rosetta LR: Feature selection was applied using mutual information prioritization with resistance phenotype. For Rosetta LR, additional preprocessing included averaging across protein subunits and removal of features with absolute pairwise correlation >0.8 before mutual information ranking.

Three neural network models (MLP, BRNN, and CNN) were implemented using R (v3.6.1), Keras (v2.11.1), and TensorFlow (v2.12.0), following Steiner et al.^25^ Input sequences were integer-encoded; class weights were used to address imbalance, and models were trained for 500 epochs using RMSprop with batch size 64 and binary cross-entropy loss (Table 1).

The MLP architecture combined embedding and global average pooling with four hidden layers (33 units, ReLU), L2 regularization, and sigmoid output. The BRNN included one bidirectional LSTM layer (33 units, 0.2 dropout) with a sigmoid output. The CNN employed two 1D convolutional layers (32 filters, kernel size of 9), one max pooling layer, and sigmoid output.

The architectures were replicated without modification from the original studies to ensure benchmark consistency. This design isolates the impact of data set construction by removing architectural variability.

RF and KNN classifiers were implemented in Python (v3.10.8) using scikit-learn (v1.5.2), following the approach of Shen et al.^28^ (Table 1). We employed Delaunay triangulation on the 3OXC^50^ crystal structure to extract essential interactions among Cα atoms, forming a graph of residue pairs with direct spatial interactions that summarizes the protein structure. We computed the feature vector by summing distances along each arc connecting amino acids of the same type, which condensed the sequence and structure information into a compact 210-element vector (representing all possible pairwise combinations of the 20 standard amino acids). We applied K-nearest neighbor and RF algorithms to train learning models using these 210-dimensional vectors coupled with phenotypic data. We set *K* = 6 for KNN and used 10 subtrees for RF, implementing both algorithms using Python v3.10.8 and scikit-learn v1.5.2.

### zScales and Rosetta-Based Logistic Regression

In addition to replicating established models, we developed two new logistic regression models with distinct feature representations to evaluate whether physicochemically informed models could maintain predictive performance while offering improved interpretability. Unlike the benchmark models, these required full development and optimization. Both models used L1 regularization and class weighting to address imbalance, with regularization strength (C) optimized via 5-fold cross-validation. After optimization, we selected the configuration that achieved the highest cross-validation performance on training data for the final evaluation. Model parameters are summarized in Table 1.

For the first model (zScales LR), we implemented zScales descriptors,^41^ where each amino acid position was represented by five sets of physicochemical properties (lipophilic (z1), steric/polarizability (z2), polarity (z3), electronegativity, heat of formation, electrophilicity, and hardness (z4, z5)), resulting in a 495-dimensional feature vector for each 99-residue protease sequence. These descriptors were selected for their comprehensive representation of amino acid properties relevant to protein–drug interactions. While dimensionality reduction techniques (e.g., PCA) were considered, we retained the full feature set to preserve the biological interpretability of the model, as each feature directly corresponds to a specific position and physicochemical property.

For the Rosetta LR model (Table 1), we employed the HIV PR crystal structure (PDB: 3OXC^50^) as a template, with water molecules and ligands removed, followed by three rounds of energy minimization using the pyRosetta v4.2 FastRelax protocol.^51^ We modeled each variant with PyRosetta’s repacking function to optimize rotamers of mutant and neighboring residues, followed by an additional round of FastRelax minimization. We extracted energetic terms for each residue by utilizing the REF2015 scoring function. Given the dimeric nature of the structure, the initial feature vector contained 3763 elements (19 terms × 99 residues × 2 subunits + Δ*G*). We averaged values over subunits using NumPy v1.24.4, resulting in 1882 features, followed by scaling all features to the [0,1] range. We then filtered out features exhibiting pairwise absolute correlations exceeding 0.8 to reduce the multicollinearity. For feature selection, we computed residue-wise mutual information between the remaining features and resistance phenotypes using scikit-learn version 1.5.2, ranking features by their mutual information values to identify the most informative predictors of PI resistance. This filtering process retained an average of 1487 ± 37 features across all drug-data set combinations (range: 1441–1531), representing approximately 79% of the original 1882 REF2015 energy terms. All retained features were used for model training (detailed breakdown by drug and data set in Table S1).

### Performance Metrics

For each evaluation step, accuracy, precision, recall, and Area Under the Receiver Operating Characteristic Curve (AUC) values were recorded using scikit-learn v1.5.2. We used the test data set to calculate AUC values for method comparisons. The AUC served as our primary comparison metric due to its ability to effectively capture performance at different classification thresholds while remaining robust to class imbalance.^52^

### Computational Performance Analysis

We evaluated the computational efficiency of each model by measuring the prediction time for a single HIV-1 protease sequence against NFV resistance. All measurements were conducted on an AMD EPYC 7662 64-Core Processor using standardized computing resources (1 CPU core, 10 GB RAM) allocated through the SLURM workload manager. Each timing measurement included the complete prediction pipeline: model loading, sequence preprocessing from a FASTA file, feature extraction, and final prediction output. For neural network models (MLP, CNN, and BRNN), this involved integer sequence encoding and prediction. KNN and RF models required Delaunay triangulation on HIV-1 protease crystal structure coordinates (PDB: 3OXC^50^) and 210-dimensional feature vector generation. The zScales LR model involved physicochemical descriptor calculation, while Rosetta LR required structure modeling, energy minimization, and REF15 energy term extraction. All Rosetta calculations were performed using CPU-only processing without GPU acceleration, as pyRosetta v4.2 was configured for single-threaded CPU execution in our computational environment. To ensure the measurement stability, we ran each model 100 times. This standardized testing environment allowed us to directly compare the computational requirements between different models under realistic deployment conditions.

### Mutual Information and Correlation Analysis

To evaluate how different sequence processing procedures affect data consistency and feature representation, we calculated the correlation between residue-wise normalized mutual information values in the NFV data set across the Steiner, Shen, and In-house data sets. We employed two distinct feature representation methods: zScales descriptors and Rosetta energy terms. Mutual information was computed with scikit-learn v1.5.2, quantifying the dependency between each amino acid position and the resistance phenotype. Before correlation analysis, we normalized them to the [0, 1] range. Pairwise correlations between data sets were calculated via the Pearson correlation coefficient (*r*), with statistical significance determined by *p*-value calculations. We performed the computational analysis using Python version 3.10.8, conducting statistical computations through the scikit-learn and NumPy libraries.

### Structures Visualization

We visualized the structures using ChimeraX v 1.3. We prepared all figures using Matplotlib v3.9.

## Results and Discussion

### Data Set Construction

We analyzed three data sets derived from the Stanford HIV Drug Resistance Database genotype–phenotype data, comprising 4504 with PIs susceptibility data. We constructed these data sets using distinct preprocessing strategies, which led to different final compositions.

The Shen’s data set employed sequence expansion at ambiguous positions, generating multiple sequences with identical resistance values. This preprocessing strategy generated 500,390 total records from 2096 unique original sequences, resulting in 99.58% of the data set consisting of expanded copies (498,294 records), of which 151,560 (30%) showed resistance to at least one inhibitor. Individual drug subsets ranged from 475,184 sequences for darunavir (DRV) to 500,205 sequences for NFV, with resistance proportions varying from 3% for DRV to 25% for NFV (Table 2). No drug showed a balanced distribution with susceptible sequences consistently representing the majority. The most severe imbalances were observed in second-line PIs, with DRV showing only 3% resistant sequences and TPV showing 10%. Creating multiple identical sequences likely introduces significant redundancy and autocorrelation within the data, as it is impossible to determine the true strain sequences present in the biological sample.^27^

**Table 2:** Distribution of Resistant and Susceptible Sequences across HIV-1 PI Data Sets

	Steiner’s data set (Steiner, 2020)			Shen’s data set (Shen, 2016)			In-house data set		
Drug	total	resistant (*n*)	resistant proportion	total	resistant (*n*)	resistant proportion	total	resistant (*n*)	resistant proportion
FPV	1415	522	0.369	498,750	84,472	0.169	815	303	0.372
IDV	1461	641	0.439	499,420	123,493	0.247	839	396	0.472
NFV	1502	794	0.529	500,205	125,668	0.251	864	505	0.584
SQV	1453	548	0.377	499,456	90,876	0.182	849	344	0.405
ATV	972	424	0.436	490,593	117,769	0.240	581	256	0.441
LPV	1248	562	0.450	497,699	62,659	0.126	703	276	0.393
DRV	599	130	0.217	475,184	12,920	0.027	372	61	0.164
TPV	690	124	0.180	476,622	48,987	0.103	412	112	0.272
total sequences	1540	851	0.553	500,390	151,561	0.303	869	529	0.609

Steiner’s data set consisted of 1540 unique sequences, with 851 (55%) labeled as resistant. The number of sequences varied considerably by drug, ranging from 599 sequences for DRV to 1502 for NFV. Resistance proportions showed substantial variation, from 18% for TPV to 53% for NFV. While their ambiguous sequence treatment method is more conservative than full sequence expansion, this method still has an important limitation, as the resulting sequences, created by arbitrarily selecting amino acids at ambiguous positions, may represent artificial combinations that do not exist in nature.

Our In-house data set implemented the most stringent preprocessing method by excluding sequences with ambiguous positions. After filtering identical sequences and keeping the median fold resistance value for each unique variant, we obtained 869 unique sequences, of which 529 (61%) were classified as resistant. Individual drug subsets ranged from 372 sequences for DRV to 864 for NFV, with resistance proportions varying from 16% for DRV to 58% for NFV.

Between the three data sets, a common issue is the scarcity of data, particularly for second-line PIs. While the Stanford HIV Drug Resistance Database represents the largest publicly available resource, newer drugs like DRV and TPV have significantly fewer sequences with matched phenotypic data compared to first-line therapies.^25,38^ This data scarcity is further complicated by substantial class imbalance in all data sets, with resistant sequences typically the minority class. The imbalance is particularly pronounced for second-line therapies.

### Test Data Set Construction and Characteristics

We evaluated three strategies for constructing HIV-1 PI resistance test data sets. Shen’s data set employed an 80:20 random split after sequence expansion of ambiguous positions, producing the most extensive test sets (95,047–100,041 sequences) with relatively low proportions of resistant sequences (0.027–0.252) (Table 3). Despite the greater sequence count, expansion increases the difficulty of random training/test splitting, where sequences expanded from one sequence, which are highly similar, appear in both training and test data, losing the independence of both data sets.^27^ The Steiner data set 80:20 random split yielded moderate-sized test sets (120–300 sequences) with resistant proportions ranging from 0.175 to 0.520.

In our In-house protocol, test set construction prioritized sequence diversity through cluster-based sampling, as illustrated in Figure 1 by using NFV data. NFV was selected for its comparatively larger sample size (Steiner’s: *n* = 1502; Shen’s: *n* = 500,206; and In-house: *n* = 864), which enabled robust statistical analysis and diverse clustering to assess how data set size and composition influence resistance predictions. Following *t*-SNE dimensionality reduction, the visualization of fold change values revealed distinct resistance patterns that aligned with sequence-based grouping (Figure 1A). K-means analysis identified five distinct sequence clusters (Figures 1C and S2) that showed significant association with resistance phenotypes.

**Figure 1:**
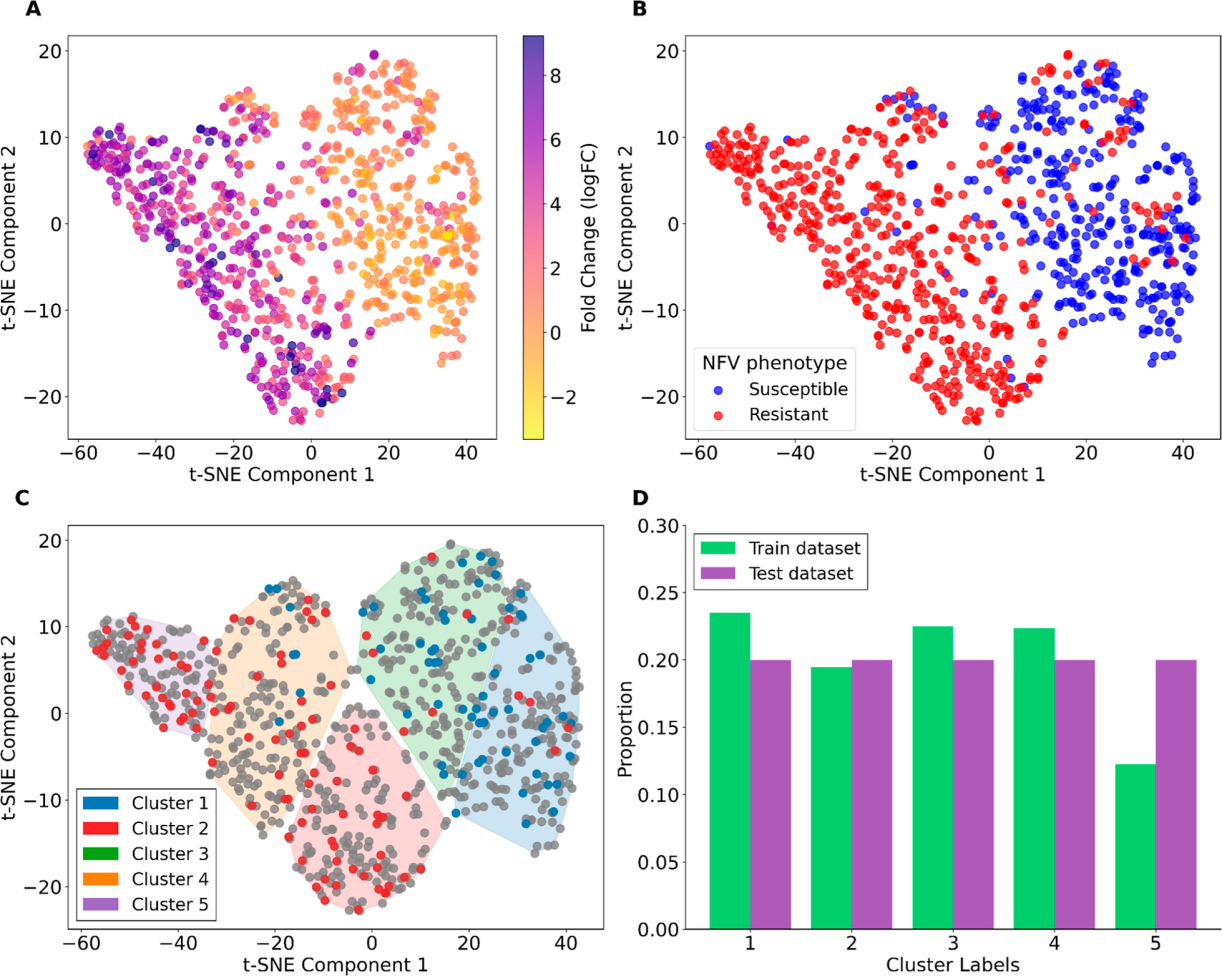
Visualization and analysis of HIV-1 protease sequence diversity using *t*-SNE dimensionality reduction. (A) *t*-SNE projection colored by fold change values, revealing the continuous spectrum of resistance levels. (B) Distribution of NFV resistance phenotypes through sequence space, with points colored by resistant (red) or susceptible (blue) status. (C) *t*-SNE projection of K-means results (5 clusters shown in different colors) with test set samples highlighted (resistant in red, susceptible in blue). Gray points represent training set sequences. Colored shading indicates cluster boundaries. (D) Comparison of cluster proportions between training (green) and test (purple) data sets, demonstrating balanced sampling among the five sequence groups.

Mapping resistance phenotypes onto the *t*-SNE projection demonstrated clear segregation patterns, with resistant (red) and susceptible (blue) sequences showing preferential aggregation based on sequence similarity (Figure 1B), consistent with Pikalyova et al. (2022).^1^ Statistical analysis showed a strong association between sequence-based clusters and resistance levels, with both one-way ANOVA (*F*_4_,_859_ = 277.49, *p* = 4.59 × 10^–153^) and Kruskal–Wallis tests (*H* = 508.74, *p* = 8.63 × 10^–109^) confirming highly significant differences in mean log_10_(fold change) across groups (Figure 1A and Table S3). The clusters displayed clear differences in the resistance profiles. Groups 1 and 3 consisted mostly of susceptible sequences, with only 12.2% and 25.8% resistance and low mean log_10_(FC) values (0.096 and 0.268). In contrast, Groups 2, 4, and 5 were strongly associated with resistance, showing 93.5%, 93.7%, and 99.2% resistance rates alongside higher mean log_10_(FC) values (1.267, 1.329, and 1.604) (Table S2).

To evaluate model generalizability and ensure performance metrics would reflect the ability to predict resistance throughout the full spectrum of sequence diversity rather than just dominant patterns, we implemented a cluster-based sampling strategy, creating test sets containing 70–170 sequences with resistant case proportions ranging from 0.171 to 0.623. For NFV, despite the imbalance with 62% resistant sequences in the test set, the strategy ensured inclusion of samples from each cluster (Figure 1D), allowing assessment of the model performance over the complete sequence diversity range.

Having established these methodologically distinct test data sets, we next assessed how different preprocessing strategies influenced model performance evaluation across various machine learning models.

### Comparative Analysis of Data Sets for HIV-1 PI Resistance Modeling

Our evaluation of seven machine learning models across three data sets—Steiner’s, Shen’s, and In-house—revealed distinct patterns in prediction performance (as measured on the 20% held-out test sets) according to the data sets’ characteristics (Figure 2). The more balanced data sets (Steiner’s and In-house) exhibited greater performance variability. In contrast, Shen’s data set exhibited uniformly high performance across all models and drugs (Table 4).

**Figure 2:**
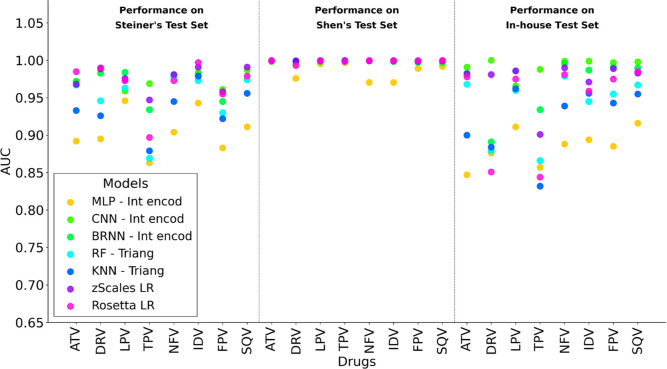
Performance of all tested classifiers per drug in Steiner’s,^25^ Shen’s,^28^ and In-house data sets. Area Under the ROC Curve (AUC) values calculated on test sets for seven different models (MLP, CNN, BRNN, RF, KNN, zScales LR, and Rosetta LR) across eight PIs. Values represent the performance per drug and method on the 20% held-out test data sets generated by each construction strategy.

**Table 3:** Composition of Test Data Sets for HIV-1 PI Resistance Prediction

	Steiner data set (Steiner, 2020)			Shen data set (Shen, 2016)			In-house data set		
drug	total	resistant (*n*)	resistant proportion	total	resistant (*n*)	resistant proportion	total	resistant (*n*)	resistant proportion
FPV	283	109	0.385	99,750	17,034	0.171	160	64	0.400
IDV	292	119	0.408	99,884	24,575	0.246	165	87	0.527
NFV	300	156	0.520	100,041	25,175	0.252	170	106	0.623
SQV	291	112	0.385	99,891	18,188	0.182	165	79	0.479
ATV	194	83	0.428	98,118	23,466	0.239	115	62	0.539
LPV	250	102	0.408	99,540	12,417	0.125	140	57	0.407
DRV	120	21	0.175	95,047	2533	0.027	70	12	0.171
TPV	138	31	0.225	95,325	9682	0.102	80	22	0.275

In Shen’s data set, all models achieved high performance with minimal variation among them (Figure 2). The RF achieved near-perfect mean metrics (AUC: 0.999, accuracy: 0.999, and precision: 0.998), followed closely by KNN (AUC: 0.999, accuracy: 0.998, and precision: 0.999) and both logistic regression (zScales LR and Rosetta LR—AUC: 1.000 and 0.999). The neural network architectures also showed excellent performance, with CNN (AUC: 0.998, accuracy: 0.998, and precision: 0.989) and BRNN (AUC: 0.999, accuracy: 0.999, and precision: 0.993) achieving near-perfect metrics. Even the simpler MLP architecture maintained high mean performance (AUC: 0.986, accuracy: 0.982, and precision: 0.901). The generation of multiple identical sequences with shared resistance values likely introduces substantial redundancy within the data since it is impossible to determine the true strain sequences present in the biological sample.^27^ This redundancy appears to facilitate model memorization rather than true generalization. This is particularly evident when comparing performance across data sets (Table 4). While models achieved near-perfect metrics on Shen’s expanded data set, their performance dropped significantly when evaluated on the other data sets.

**Table 4:** Mean Performance Metrics (± Standard Deviation) Averaged across Eight HIV-1 PIs for Machine Learning Models on Three Data Sets (Calculated on 20% Held-Out Test Sets)

	accuracy			precision			recall			AUC		
	Steiner	Shen	In-house	Steiner	Shen	In-house	Steiner	Shen	In-house	Steiner	Shen	In-house
MLP	0.838 ± 3.21 × 10^–2^	0.982 ± 1.22 × 10^–2^	0.807 ± 2.38 × 10^–2^	0.763 ± 1.44 × 10^–1^	0.901 ± 1.07 × 10^–1^	0.825 ± 1.68 × 10^–1^	0.783 ± 5.41 × 10^–2^	0.954 ± 4.83 × 10^–2^	0.659 ± 8.48 × 10^–2^	0.905 ± 2.85 × 10^–2^	0.986 ± 1.19 × 10^–2^	0.884 ± 2.40 × 10^–2^
CNN	0.922 ± 2.79 × 10^–2^	0.998 ± 9.19 × 10^–4^	0.970 ± 2.19 × 10^–2^	0.891 ± 4.81 × 10^–2^	0.989 ± 7.02 × 10^–3^	0.956 ± 6.06 × 10^–2^	0.886 ± 8.71 × 10^–2^	0.997 ± 2.55 × 10^–3^	0.888 ± 2.93 × 10^–2^	0.975 ± 1.14 × 10^–2^	0.998 ± 1.33 × 10^–3^	0.992 ± 1.14 × 10^–2^
BRNN	0.919 ± 3.03 × 10^–2^	0.998 ± 1.47 × 10^–3^	0.927 ± 4.33 × 10^–2^	0.867 ± 7.91 × 10^–2^	0.995 ± 5.03 × 10^–3^	0.876 ± 1.47 × 10^–1^	0.891 ± 7.78 × 10^–2^	0.995 ± 3.10 × 10^–3^	0.812 ± 9.02 × 10^–2^	0.970 ± 1.92 × 10^–2^	0.999 ± 2.10 × 10^–3^	0.970 ± 3.74 × 10^–2^
RF	0.892 ± 4.55 × 10^–2^	0.999 ± 3.35 × 10^–4^	0.880 ± 4.39 × 10^–2^	0.839 ± 8.87 × 10^–2^	0.998 ± 5.68 × 10^–4^	0.835 ± 1.39 × 10^–1^	0.790 ± 2.20 × 10^–1^	0.996 ± 3.39 × 10^–3^	0.777 ± 2.12 × 10^–1^	0.950 ± 3.63 × 10^–2^	0.999 ± 1.76 × 10^–4^	0.940 ± 4.29 × 10^–2^
KNN	0.866 ± 4.91 × 10^–2^	0.998 ± 4.58 × 10^–4^	0.835 ± 4.18 × 10^–2^	0.907 ± 5.30 × 10^–2^	0.999 ± 7.36 × 10^–4^	0.930 ± 8.97 × 10^–2^	0.670 ± 2.19 × 10^–1^	0.990 ± 6.48 × 10^–3^	0.608 ± 2.07 × 10^–1^	0.939 ± 3.18 × 10^–2^	0.999 ± 4.08 × 10^–4^	0.921 ± 4.57 × 10^–2^
zScales LR	0.905 ± 4.92 × 10^–2^	0.994 ± 1.89 × 10^–3^	0.907 ± 3.78 × 10^–2^	0.866 ± 1.46 × 10^–1^	0.973 ± 5.39 × 10^–2^	0.869 ± 1.13 × 10^–1^	0.919 ± 2.26 × 10^–2^	0.987 ± 2.36 × 10^–3^	0.889 ± 6.74 × 10^–2^	0.984 ± 1.61 × 10^–2^	0.999 ± 1.09 × 10^–4^	0.973 ± 2.96 × 10^–2^
Rosetta LR	0.926 ± 2.23 × 10^–2^	0.995 ± 3.00 × 10^–3^	0.866 ± 5.68 × 10^–2^	0.890 ± 7.94 × 10^–2^	0.969 ± 2.61 × 10^–2^	0.780 ± 1.92 × 10^–1^	0.911 ± 5.60 × 10^–2^	0.911 ± 1.07 × 10^–2^	0.907 ± 1.38 × 10^–1^	0.983 ± 3.15 × 10^–2^	0.999 ± 2.31 × 10^–3^	0.944 ± 5.98 × 10^–2^

In Steiner’s data set, performance differences between models became more apparent (Table 4). CNN showed the highest performance (AUC: 0.975, accuracy: 0.922, and precision: 0.891), followed closely by BRNN (AUC: 0.970, accuracy: 0.919, and precision: 0.867). The logistic regression models maintained high performance (zScales LR AUC: 0.975; Rosetta LR AUC: 0.969), while RF (AUC: 0.950) and KNN (AUC: 0.939) showed slightly lower metrics. MLP demonstrated the most variable performance (AUC: 0.905, accuracy: 0.838, and precision: 0.763), particularly for second-line inhibitors.

The In-house data set presented the greatest performance variability (Mean AUC range: 0.884 to 0.992), particularly for newer PIs (Figure 2). The clustering-based strategy created a more challenging test set than random division, leading to lower performance metrics and better evaluation of model generalization.^53,54^ Model performance decreased markedly compared to other data sets, with sophisticated architectures like CNN experiencing reduced metrics. Both logistic regression models demonstrated competitive performance in this challenging data set. The CNN achieved the highest overall performance (AUC: 0.992, accuracy: 0.970, precision: 0.956, and recall: 0.969), followed closely by the zScales LR (AUC: 0.973, accuracy: 0.907, precision: 0.869, and recall: 0.893) and BRNN (AUC: 0.970, accuracy: 0.927, precision: 0.876, and recall: 0.903). Statistical analysis using DeLong’s test (Table S5) revealed no significant differences between the top-performing models: CNN vs zScales LR (*p* > 0.05), CNN vs BRNN (*p* > 0.05), and zScales LR vs BRNN (*p* > 0.05). The Rosetta LR also showed competitive mean results (AUC: 0.944, accuracy: 0.866, precision: 0.780, and recall: 0.876), with no statistically significant difference from zScales LR and BRNN. These findings suggest that physicochemically informed feature representations (amino acid descriptors for zScales and energy terms for Rosetta) can achieve performance statistically equivalent to sequence-based representations used in neural network models.

While neural network architectures demonstrated strong predictive capability, with CNN and BRNN underscoring their ability to capture intricate patterns in biological sequence data,^30,55^ their complexity raises concerns with limited training samples.^26^ Model complexity is critical when few samples per class are available, with simpler architectures often outperforming complex ones in such scenarios.^56^ This is particularly evident for second-line inhibitors such as DRV and TPV, where severe class imbalance challenges neural networks models despite regularization strategies (Figure 2).

Our statistical analysis confirms that triangulation-based KNN (AUC: 0.921 ± 0.046), RF (AUC: 0.940 ± 0.043), and logistic regression models (zScales LR: 0.973 ± 0.030; Rosetta LR: 0.944 ± 0.060) attained competitive AUC values despite their simpler designs. DeLong’s test revealed that RF showed no statistically significant differences from several top models (RF vs zScales LR: *p* > 0.5; RF vs BRNN: *p* > 0.5), while the logistic regression models achieved statistically equivalent performance to more complex architectures (Table S5). Even KNN, despite ranking lowest among the tested models, demonstrated solid performance given its algorithmic simplicity. This indicates that carefully selected features and appropriate model complexity can effectively match architectural sophistication for this particular prediction challenge.

The zScales descriptor-based logistic regression exhibited particularly robust performance across data sets (Figure 2), achieving AUC values ranging from 0.947 to 0.991 (Steiner’s), 0.999–0.999 (Shen’s), and 0.901–0.990 (In-house) (Table S4). This consistency across different test set compositions suggests that the model effectively captures fundamental physicochemical determinants of PI resistance through position-specific amino acid properties. The Rosetta energy function-based logistic regression also demonstrated stable performance with AUC values ranging from 0.897 to 0.997 (Steiner’s), 0.993–0.999 (Shen’s), and 0.844–0.985 (In-house) (Table S4). This broader range reflects the structural complexity of energy-based features, which capture drug-specific energetic interactions that vary across different PIs while maintaining an overall high predictive performance.

These relatively simple logistic regression models’ ability to achieve performance comparable to more complex ones while offering clear interpretability demonstrates that well-chosen features can effectively capture key information for resistance modeling.^29^ Statistical validation using DeLong’s test confirms that there are no significant performance differences between physicochemically informed models and sophisticated neural networks on our most challenging data set. In such scenarios with well-structured data and relevant features, it is observed that there is no significant difference in performance between more complex and simpler classifiers after appropriate preprocessing.^40^

### Computational Performance Analysis

The computational efficiency of resistance prediction models is crucial for their practical implementation in clinical settings.^57^ We evaluated the time required for a single sequence prediction using each model previously trained on the three data sets (In-house, Steiner, and Shen), measuring the complete computational pipeline from sequence input through feature transformation to final prediction output (Table 5 ).

**Table 5:** Prediction Time Analysis in Seconds of Machine Learning Models across Three Data Sets for HIV-1 PI Resistance Prediction

	Steiner	Shen	In-house	mean	Stdev
MLP	0.052	0.052	0.053	0.052	0.001
CNN	0.052	0.051	0.052	0.052	0.001
BRNN	0.065	0.065	0.065	0.065	0.000
RF	2.234	2.238	2.273	2.248	0.021
KNN	2.236	2.524	2.272	2.344	0.057
Rosetta LR	777.336	773.830	777.184	776.117	1.982
zScales LR	0.006	0.007	0.007	0.007	0.000

**Table 6:** Top 10 Model Coefficients Associated with HIV-1 PI Resistance for Rosetta Energy Terms and zScales Descriptors

Rosetta			zScales		
AA-feature	coefficient	*P*-value	AA-feature	coefficient	*P*-value
24-fa_dun	22.077	2.08 × 10^–2^	30–5	5.123	0.886
52-rama_prepro	16.833	9.04 × 10^–2^	71–2	–1.885	0.971
45-fa_dun*	11.998	5.52 × 10^–3^	48–3	–1.427	0.073
84-fa_atr*	11.476	3.63 × 10^–6^	10–5	–1.380	0.171
54-fa_dun*	10.493	3.89 × 10^–2^	84–1	1.349	0.937
46-cart_bonded*	–10.154	2.77 × 10^–3^	74–1	1.269	0.222
54-lk_ball_wtd*	9.989	3.56 × 10^–2^	88–4	–1.224	0.823
52-fa_atr	–8.500	3.28 × 10^–1^	54–1	1.051	0.957
53-lk_ball_wtd	–6.482	2.60 × 10^–1^	73–5	0.919	0.996
10-fa_intra_sol_xover4*	6.017	4.67 × 10^–2^	82–3	0.884	0.842

*p-value < 0.05.

The zScales logistic regression model demonstrated the fastest prediction times, averaging 0.007 s per sequence, followed by MLP and CNN (both averaging 0.052 s) and BRNN (0.065 s). These rapid prediction times make these models suitable for clinical applications.

The traditional machine learning models showed moderate computational demands, with RF and KNN requiring approximately 2.2 s per prediction. This increased computation time likely stems from the Delaunay triangulation feature extraction process.^28^

The Rosetta LR model exhibited significantly longer prediction times, averaging 776.117 s per sequence. This considerable computational cost is primarily attributed to the structural modeling and energy calculations required to compute the energy terms for each sequence variant. Although computationally demanding for rapid point-of-care applications, it serves an important research function by providing valuable structural insights into resistance mechanisms through energy terms. Additionally, the Rosetta-based framework represents a significant computational improvement compared to conventional structural analysis methods such as molecular dynamics simulations, which typically require several hours to days of computation time per structure.^24,58,59^ Therefore, while less suitable for immediate real-time applications, the Rosetta LR model remains viable for clinical implementation, particularly when balanced against the mechanistic insights provided by structure-based energy features for resistance mechanism research and drug development applications.

### Impact of Data Set Construction Strategies on Feature Representation

In order to evaluate how sequence processing affects data consistency and feature representation, we calculated the correlation between the residue-wise normalized mutual information values in NFV data across Steiner’s, Shen’s, and In-house data sets. Pairwise comparisons were made between all three data sets (Figure 3A–F). Our analysis revealed high correlations between In-house and Steiner data sets (*r* = 0.914, p-value: 3.07 × 10^–196^ for zScales and *r* = 0.698, p-value: 1.40 × 10^–275^ for Rosetta). The strong correlation in feature importance patterns between those filtered data sets suggests that this approach effectively preserves biologically relevant signals between them.^60^

**Figure 3:**
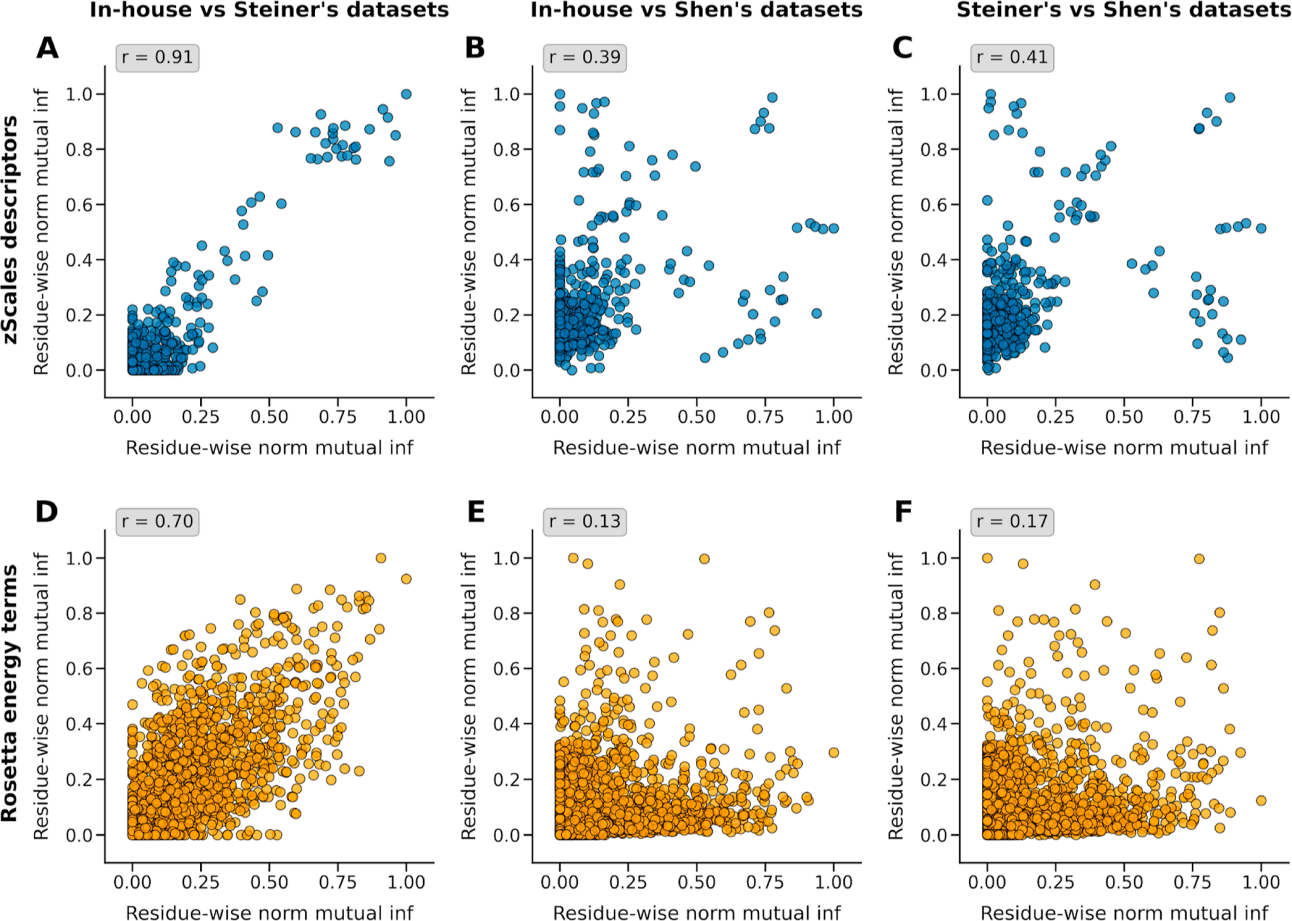
Pearson correlation between residue-wise normalized mutual information values from Nelfinavir data sets, converted to zScales amino acid descriptors ((A) In-house vs Steiner’s data sets, (B) In-house vs Shen’s data sets, and (C) Steiner’s vs Shen’s data sets) and Rosetta energy terms ((D) In-house vs Steiner’s data sets, (E) In-house vs Shen’s data sets, and (F) Steiner’s vs Shen’s data sets).

Conversely, both data sets showed low correlations with Shen’s data set, which contains a larger number of sequences due to its ambiguous sequence expansion protocol. The In-house versus Shen’s data set comparison showed a weak positive correlation (*r* = 0.388, *p*-value: 3.11 × 10^–19^ for zScales and r = 0.127, *p*-value: 3.09 × 10^–8^ for Rosetta), while Steiner’s versus Shen’s comparison demonstrated a moderate positive correlation (*r* = 0.408, *p*-value: 2.93 × 10^–21^ for zScales and *r* = 0.172, *p*-value: 5.71 × 10^–15^ for Rosetta). This suggests that Shen’s approach of expanding ambiguous sequences into multiple variants, while increasing data set size, may introduce artificial redundancy and potentially dilute genuine resistance signals. The zScales descriptors consistently demonstrated higher correlations between data sets compared to Rosetta energy terms. This stability in zScales correlations can be attributed to their position-independent physicochemical properties (lipophilicity, steric bulk, polarity, and electronic effects),^41^ whereas Rosetta energy terms depend on complex structural interactions and are sensitive to conformational changes.^35^

### zScales and Rosetta Logistic Regression Models

We developed two complementary logistic regression models to predict HIV-1 PI resistance. The first model (zScales LR) employs zScales descriptors,^41^ which transform protein sequences into numerical vectors based on five principal components, capturing fundamental physicochemical properties, including lipophilicity, steric bulk, polarity, and electronic effects. The second model (Rosetta LR) employs the Rosetta Energy Function 2015 (REF15),^35^ which approximates biomolecular conformational energetics through residue-wise calculations of physics-based and knowledge-based terms, including van der Waals interactions, solvation, and hydrogen bonding. Unlike position-specific approaches, REF15 captures local and nonlocal effects of mutations through its comprehensive evaluation of multiple energetic contributions across the protein structure. These complementary methods provide interpretable frameworks for understanding resistance mechanisms from physicochemical and structural energetics perspectives.^35,41^

The two models demonstrate high predictive performance while providing insights through interpretable coefficients. Our analysis of model coefficients reveals the relative contribution of different features to resistance prediction, with each model capturing distinct but complementary aspects of resistance mechanisms. A comparison of coefficient distributions (Table 6) shows that the Rosetta model uses fewer total parameters (51) but has a higher proportion of statistically significant coefficients (*p* < 0.05) compared to the zScales model (107 parameters), reflecting the structural specificity of the Rosetta energy function.

The mutual information analysis identifies specific amino acid positions with substantial information content related to resistance phenotypes, especially around the substrate binding site and flap regions (Figure 4). The zScales representation shows a punctate pattern, where specific amino acid positions emerge as distinct hotspots with high mutual information values. This is due to the fact that the zScales descriptors represent individual amino acid characteristics and do not include the physicochemical effects of mutations in the neighborhoods (Figure 4A). Consequently, the visualization shows well-defined, isolated positions of high information content against a background of lower values.

**Figure 4:**
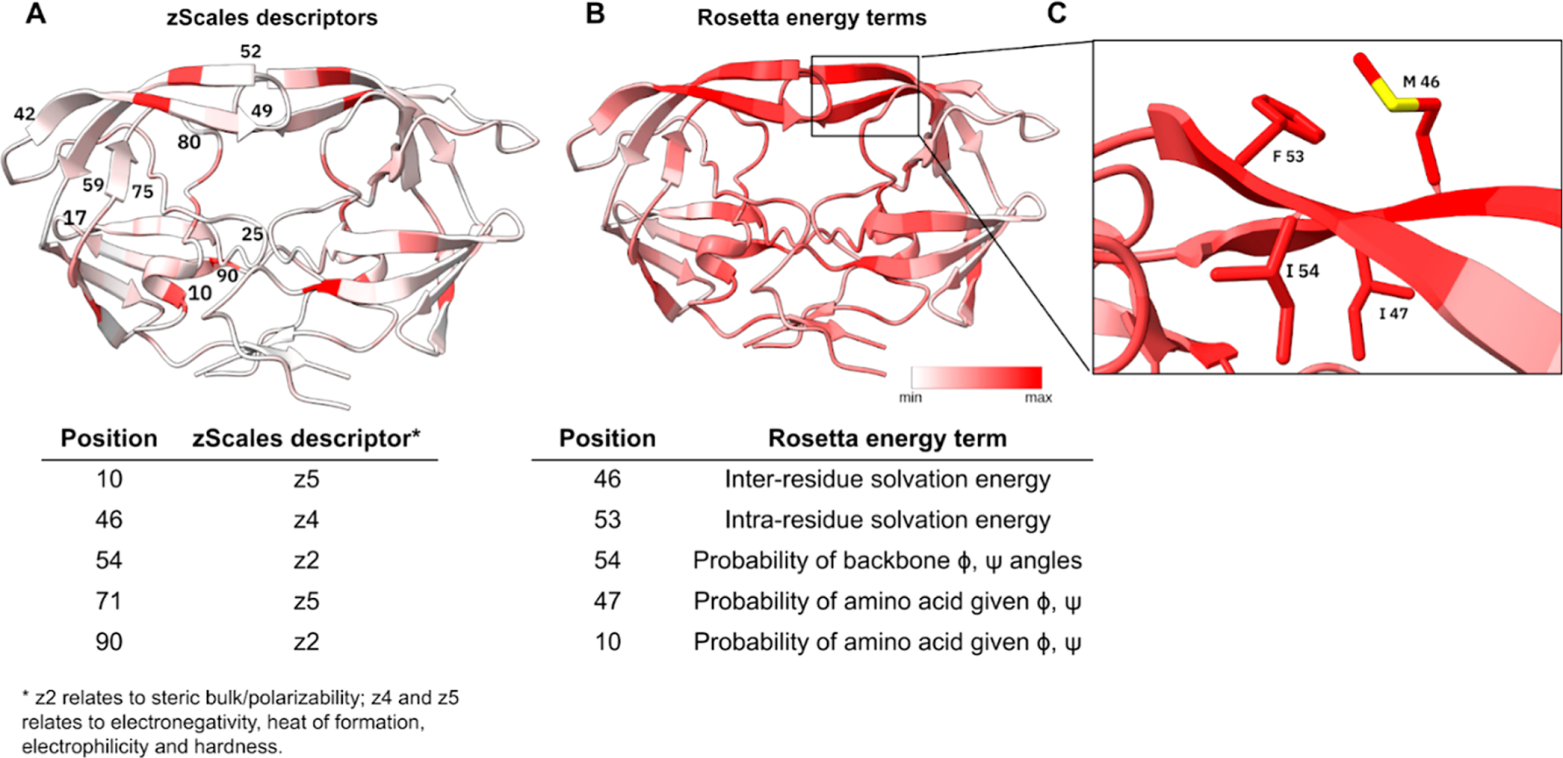
Maximum residue-wise mutual information values relating to the target per amino acid in Nelfinavir isolates converted to (A) zScales amino acid descriptors and (B) Rosetta energy terms. White represents the lowest mutual information values, and red represents the highest. (C) Residues with the highest residue-wise mutual information values in the Rosetta energy terms representation group predominantly within the flap region.

The Rosetta energy terms display a continuous pattern of mutual information values, particularly in the flap regions and around the catalytic site, where areas of high mutual information values extend across structurally adjacent residues. The highest mutual information values were observed at positions 46, 53, 54, 47, and 10, with four residues in the flap region in close steric proximity (Figure 4C) and related to drug resistance. This pattern suggests that Rosetta’s energy function captures how a single mutation influences the energetics of surrounding residues through various interactions (van der Waals, electrostatics, and hydrogen bonds), resulting in broader regions of elevated mutual information values (Figure 4B).

Our coefficient analysis further validates these findings, revealing a spectrum of energy terms that capture different aspects of the protein structure and energetics. Conformational energetics (fa_dun terms at positions 24, 45, and 54), backbone geometry (rama_prepro), solvation effects (lk_ball_wtd), structural geometry (cart_bonded), and van der Waals interactions (fa_atr) all show significant coefficients, providing a multidimensional view of resistance mechanisms.

These identified residue positions play critical roles in PI resistance through distinct structural and biochemical mechanisms. The L10F mutation alters hydrogen bonding patterns with PIs, particularly NFV.^61^ At position 10, both the z5 descriptor and the Rosetta energy term ″Probability of amino acid identity given φ, ψ angles″ capture key aspects of this resistance mechanism.

The z5 descriptor reflects the electrochemical properties that influence the altered hydrogen bonding patterns, while the amino acid probability term indicates the likelihood and impact of specific substitutions at this position. Furthermore, position 10 mutations often occur as part of cooperative networks, with Q58E serving as an accessory mutation linked to L10I, I54 V, M46L, and V82A, highlighting the interconnected nature of resistance mutations.^62^

Another relevant residue is M46, where the M46LI mutations frequently emerge after NFV therapy and typically occur in conjunction with D30N and/or L90 M mutations.^62,63^ M46 showed association with both the z4 descriptor (which captures electronic properties including electronegativity and heat of formation) and inter-residue solvation energy terms from Rosetta (Figure 4), consistent with its role in modulating hydrophobic interactions and β-sheet structure near the flap region.^64,65^ The significant negative coefficient for 46-cart_bonded (−10.154, *p* = 2.77 × 10^–3^) in our Rosetta model further demonstrates that deviations from ideal bond lengths and angles at position 46 strongly correlate with resistance, suggesting that mutations induce subtle but consequential distortions in local geometry.^35^

Positions 53 and 54 showed notable signals in both representations, with position 53 contributing to solvation-related Rosetta energy terms. Although F53 is not directly linked to drug resistance, it significantly influences protein dynamics and drug binding due to its strategic location in the flap region, where it forms van der Waals contacts with the substrate, and its proximity to the resistance-associated residue M46 ^66^ (Figure 4C). The I54 residue, characterized by the z2 descriptor (steric bulk and polarizability) and backbone φ, ψ angle probability terms from Rosetta, plays a crucial role in multidrug resistance, including NFV, IDV, and SQV.^45,67^ The z2 scale effectively captures how I54VLM mutations differently impact hydrophobic packing and van der Waals interactions—I54 V stabilizes the structure while I54L disrupts hydrophobic packing with P79, impairing flap closure.^5^ Also, the I54 V mutation forms a cooperative network with G48 V, where both mutations destabilize the flap tips conformations despite not being in direct contact.^68^ Our coefficient analysis confirms the importance of position 54, with significant values for both 54-fa_dun (10.493, *p* = 3.89 × 10^–2^) and 54-lk_ball_wtd (9.989, *p* = 3.56 × 10^–2^) terms, suggesting that both side-chain conformational preferences and solvation patterns at this position contribute to resistance.^35^

Positions 71 and 90 also showed significant mutual information in the zScales representation. Position 71, associated with the z5 descriptor (related to electronegativity and electronic properties), demonstrates how nonactive site mutations contribute to resistance through long-range effects. The A71 V mutation’s impact on electronic properties, captured by z5, helps explain how this distal change can propagate alterations to the catalytic aspartates through the modification of the van der Waals interaction network and ultimately affect the protease function, influencing protein dynamics and stability from its position outside the active site.^69^ L90, characterized by z2 (steric bulk and polarizability), contributes to this network of resistance mutations. The L90 M mutation introduces interactions with the catalytic D25 and D25′absent in the wild-type enzyme.^69^ These altered interactions, linked to z2’s focus on steric properties, disrupt the catalytic site, impacting inhibitor/substrate binding and catalysis. Combined with decreased dimer stability and altered catalytic activity, this explains L90M’s role in cross-resistance to most clinical PIs. Collectively, these positions highlight how mutations at nonactive sites can confer resistance through long-range perturbations of catalytic residues, with their effects effectively captured by distinct physicochemical properties in the zScales descriptor system.^69^

Mapping significant coefficients onto the HIV-1 protease structure reveals distinct spatial clusters corresponding to known functional regions. The flap region (positions 45–54), substrate binding pocket (positions 24, 30, 82, and 84), and distal sites (positions 10, 71, and 90) all show significant coefficients in one or both models, highlighting the comprehensive structural basis of resistance captured by our method. The distribution of these positions across the protein structure reveals distinct categories of resistance mutations. Some positions (46, 82, and 84) represent active site mutations that directly alter protease–inhibitor interactions, while others (54, 90) are distal mutations that transmit conformational changes to the active site cavity.^4^

The zScales descriptors and Rosetta energy terms provide different views into HIV-1 PI resistance mechanisms. The zScales descriptors identify specific resistance hotspots through position-specific physicochemical properties, showing discrete positions in which mutations impact drug binding or protein function. In contrast, Rosetta’s energy-based representation considers structural context, with energy terms reflecting contributions from local and nonlocal interactions in functional regions like the flaps. While zScales offers precise localization of key positions, its limitation to single-residue properties highlights the value of structure-based methods such as Rosetta that can capture broader structural impacts of mutations. These distinct approaches illustrate how feature representation choices influence our ability to analyze resistance mechanisms on different scales. This framework has potential applications beyond HIV, including resistance modeling in other viral proteins. The models’ interpretability makes it particularly suitable for clinical implementation, where understanding the basis of predictions is crucial.^40^ Furthermore, insights from physicochemical and structural features can inform drug development strategies.^24^

### Limitations and Perspectives

The Rosetta LR model’s computational cost (776.117 s per prediction) renders it less suitable for instantaneous point-of-care clinical applications where rapid treatment decisions are essential. While Rosetta provides valuable mechanistic insights for resistance research and drug development, its practical clinical utility is severely limited by computational demands. This limitation highlights the clinical advantage of the zScales LR model, which achieves comparable predictive performance while providing near-instantaneous predictions (0.007 s). The limited availability of high-quality resistance data for newer PIs such as darunavir and tipranavir further constrains our models.^38^ Although we employed weighting strategies to mitigate class imbalances, these data limitations may still impact model performance, particularly for drugs with severely imbalanced data sets.^25^

This study focuses on HIV-1 subtype B and analyzes resistance exclusively on the basis of protease mutations, which restricts the generalizability of our findings to other viral subtypes and does not account for variations in other genomic regions that may influence resistance outcomes. Our models are trained exclusively on *in vitro* phenotypic data, which allows us to capture the direct relationship between protease mutations and drug susceptibility without confounding factors. However, for clinical applications, additional considerations would be necessary, including patient-specific variables (adherence, pharmacokinetics, and drug interactions), host factors, and mutations in other viral regions, which are beyond the scope of this structure-based resistance prediction approach.^32,70^

Including diverse HIV-1 subtypes could improve global applicability and enhance predictive performance. Expanding this methodology to encompass resistance modeling across multiple viral targets (protease, reverse transcriptase, and integrase) would better reflect the complexity of combination antiretroviral therapies used clinically. Our computational framework could be adapted for other rapidly mutating viruses such as HCV or SARS-CoV-2, where drug resistance presents similar challenges. This adaptation would necessitate the recalibration of model parameters to accommodate virus-specific mutation patterns, the incorporation of appropriate structural templates for feature extraction, and the integration of pathogen-specific resistance threshold values derived from clinical data. Beyond clinical applications, our findings offer valuable insights into drug discovery. By identifying critical physicochemical properties and structural features contributing to resistance, such as electronic properties affecting hydrogen bonding networks, side-chain conformational energetics, solvation patterns at the flap regions, and local geometric distortions, it is possible to design novel inhibitors that are effective against resistant variants. Understanding the mechanistic basis of resistance mutations enables the development of compounds that anticipate and overcome common resistance pathways.

Our benchmarking framework could extend beyond viral resistance modeling to diverse scientific applications. In medical sciences, physicochemically informed models can be used in pharmacogenomics and real-time clinical decision systems, which is an important topic in drug discovery and development.^71–74^ Similar initiatives can be carried out in environmental applications and computational geoscience, and clustering-based validation and class imbalance handling can potentially be used to improve pollution prediction and monitoring systems.^75–83^ Future work should focus on unified cross-domain frameworks and their integration with emerging technologies.

## Conclusion

This study presents a comprehensive benchmarking analysis of machine learning models for predicting HIV-1 PI resistance and systematically evaluates how data set construction impacts model performance. Our results demonstrate that data preprocessing strategies, particularly sequence expansion methods, can artificially inflate performance metrics due to embedded redundancy. While all tested models achieved near-perfect classification (mean AUC: 0.986–0.999) on expanded data sets like Shen’s, their performance significantly diverged when evaluated on more rigorously curated data sets employing cluster-based sampling. The CNN achieved the highest overall performance on our rigorous In-house data set (mean AUC: 0.992), followed closely by the zScales logistic regression model (mean AUC: 0.973), which demonstrated competitive accuracy comparable to complex neural networks architectures while offering superior computational efficiency (0.007 s per sequence) and interpretability. Statistical analysis using DeLong’s test revealed no significant differences between top-performing models (*p* > 0.05), confirming that physicochemically informed feature representations can achieve performance statistically equivalent to sequence-based neural network approaches.

Feature analysis revealed distinct yet complementary resistance signatures: zScales descriptors identified key mutation hotspots through position-specific physicochemical properties (positions 10, 46, 54, 71, and 90), while Rosetta energy terms provided a holistic view of resistance mechanisms by quantifying destabilizing effects across structural regions, particularly in the flaps (residues 46–54) and catalytic site. This dual perspective advances our mechanistic understanding of resistance, bridging sequence-based, and structure-based paradigms.

Several limitations warrant consideration. First, the computational demands of Rosetta-based predictions (776.117 s per sequence) hinder real-time clinical deployment. Second, limited training data for newer inhibitors (e.g., DRV and TPV) restrict model generalizability for these compounds. Third, our focus on HIV-1 subtype B may not capture resistance patterns in other globally prevalent subtypes. Finally, incorporating additional biological context, such as host factors or viral replication fitness, could improve the predictive accuracy.

Looking forward, this work establishes a framework for future resistance modeling studies. Key directions include integrating longitudinal clinical data to model resistance evolution dynamically, expanding training data to encompass diverse HIV-1 subtypes and newer drug classes and extending this approach to other antiviral targets. The physicochemical and structural insights gleaned here may also inform rational drug design strategies to combat resistance, particularly in targeting conserved regions that are resilient to mutation. By combining interpretable machine learning with biophysical feature engineering, this study provides practical tools for resistance prediction and fundamental insights into viral adaptation mechanisms.

## Supplementary Material



## Data Availability

The input data used in this study are available in the Zenodo repository: 10.5281/zenodo.14926801. The codes used in this study are available in the GitHub repository: https://github.com/riveros94/hiv_pr_benchmarks. The coefficients of Rosetta and zScales LR models are available in the Zenodo repository: https://zenodo.org/records/15399541

## Notes

Detailed feature selection for the Rosetta LR model by data set and drug; descriptive statistics of nelfinavir (NFV) clustering; statistical analysis of clustering in the nelfinavir (NFV) data set; performance metrics for ATV, DRV, FPV, IDV, LPV, NFV, SQV, and TPV resistance prediction across all models and data sets; pairwise model comparisons using DeLong’s test; model coefficients for the NFV Rosetta logistic regression model trained using the In-house data set; model coefficients for the NFV Rosetta logistic regression model trained using the In-house data set; and optimization of *t*-SNE parameters for cluster analysis of HIV-1 protease sequences (PDF)

## Abbreviations

ATV: Atazanavir
AUC: Area Under the Receiver Operating Characteristic Curve
BLOSUM80: Blocks Substitution Matrix 80
BRNNs: Bidirectional Recurrent Neural Networks
BRNN: Bidirectional Recurrent Neural Network
Cα: α Carbon
CNN: Convolutional Neural Network
CNNs: Convolutional Neural Networks
CPU: Central Processing Unit
DRM: Drug Resistance Mutation
DRV: Darunavir
Δ*G*: Change in Gibbs Free Energy
FPV: Fosamprenavir
HCV: Hepatitis C Virus
HIV-1: Human Immunodeficiency Virus type 1
IC50: Half-maximal Inhibitory Concentration
IDV: Indinavir
KNN: k-Nearest Neighbors
LPV: Lopinavir
LR: Logistic Regression
LSTM: Long Short-Term Memory
MLP: Multilayer Perceptron
MLPs: Multilayer Perceptrons
NFV: Nelfinavir
PCA: Principal Component Analysis
PDB: Protein Data Bank
PIs: Protease Inhibitors
PLS: Partial Least Squares
PR: Protease
*r*: Pearson Correlation Coefficient
RAM: Random Access Memory
REF15: Rosetta Energy Function 2015
REF2015: Rosetta Energy Function 2015
ReLU: Rectified Linear Unit
RF: Random Forest
SARS-CoV-2: Severe Acute Respiratory Syndrome Coronavirus 2
SLURM: Simple Linux Utility for Resource Management
SQV: Saquinavir
TPV: Tipranavir
*t*-SNE: t-Distributed Stochastic Neighbor Embedding
