# Supplementary material for: Benchmarking Machine Learning Models for HIV‑1 Protease Inhibitor Resistance Prediction: Impact of Data Set Construction and Feature Representation
Source: J Chem Inf Model. 2025 Sep 25;65(19):10037–53. doi: 10.1021/acs.jcim.5c01544 (PMC12529765; doi:10.1021/acs.jcim.5c01544)
Supplement: Supplementary file 1 [file ci5c01544_si_001.pdf]

Supporting Information

Benchmarking Machine Learning Models for HIV-1 Protease Inhibitor Resistance Prediction: Impact of Dataset Construction and Feature Representation

Rocío Lucía Beatriz Riveros Maidana[1,2]; Lucas de Almeida Machado[3*]; Ana Carolina Ramos Guimarães[1,2]

[1] Laboratório de Genômica Aplicada e Bioinovações - Instituto Oswaldo Cruz/Fiocruz, Rio de Janeiro, Brazil 21040-900
[2] Programa de Pós-Graduação em Biologia Computacional e Sistemas - Instituto Oswaldo Cruz/Fiocruz, Rio de Janeiro, Brazil 21040-900
[3] Institute of Technology on Immunobiologicals (Bio-Manguinhos) - Fiocruz, Rio de Janeiro, Brazil.

*Corresponding author: biolucasmachado@gmail.com, ORCID: https://orcid.org/0000-0002-6575-1687

1 Supplementary Tables

Table S1: **Detailed feature selection for Rosetta LR model by dataset and drug.**

**Dataset**	**Drug**	**Initial Features**	**After Corr & MI Filtering**	**Reduction (%)**
Steiner's	ATV	1.882	1.509	19.8
	DRV	1.882	1.449	23.0
	FPV	1.882	1.492	20.7
	IDV	1.882	1.499	20.4
	LPV	1.882	1.487	21.0
	NFV	1.882	1.481	21.3
	SQV	1.882	1.509	19.8
	TPV	1.882	1.442	23.4
Shen's	ATV	1.882	1.531	18.6
	DRV	1.882	1.524	19.0
	FPV	1.882	1.526	18.9
	IDV	1.882	1.526	18.9
	LPV	1.882	1.528	18.8
	NFV	1.882	1.527	18.9
	SQV	1.882	1.524	19.0
	TPV	1.882	1.517	19.4
In-house	ATV	1.882	1.449	23.0
	DRV	1.882	1.441	23.4
	FPV	1.882	1.443	23.3
	IDV	1.882	1.457	22.6
	LPV	1.882	1.457	22.6
	NFV	1.882	1.451	22.9
	SQV	1.882	1.454	22.7
	TPV	1.882	1.455	22.7

Table S2: **Descriptive statistics of nelfinavir (NFV) clustering.**

**Cluster**	**n**	**Mean $\log_{10}$(FC)**	**Std Dev**	**% Resistant**	
1	197	0.096	0.356	12.2%	
2	169	1.267	0.670	93.5%	
3	190	0.268	0.464	25.8%	
4	189	1.329	0.619	93.7%	
5	119	1.604	0.509	99.2%	
**Total**	**864**	**0.712**	**0.756**	**64.8%**	–

Table S3: **Statistical analysis of clustering in nelfinavir (NFV) dataset.**

**Statistical Test**	**Statistic**	**p-value**
One-way ANOVA	$F_{4.859} = 277.49$	4.59×10^{-153}
Kruskal-Wallis	$H = 508.74$	8.63×10^{-109}
Levene's test	$W = 22.88$	5.49×10^{-18}

Table S4: **Performance metrics for ATV, DRV, FPV, IDV, LPV, NFV, SQV and TPV resistance prediction across all models and datasets**

Drug	Method	In-house				Shen (2016)				Steiner (2020)			
		**Acc**	**Prec**	**Rec**	**AUC**	**Acc**	**Prec**	**Rec**	**AUC**	**Acc**	**Prec**	**Rec**	**AUC**
**ATV**	MLP	0.765	0.927	0.613	0.847	0.992	0.991	0.976	0.999	0.810	0.765	0.793	0.892
	CNN	0.948	0.967	0.935	0.991	0.999	0.998	0.998	0.998	0.918	0.913	0.890	0.972
	BRNN	0.939	0.966	0.919	0.983	0.999	0.999	0.998	1.000	0.903	0.880	0.890	0.972
	Triang RF	0.861	0.942	0.790	0.968	0.999	0.999	0.998	1.000	0.897	0.860	0.902	0.967
	Triang KNN	0.774	0.974	0.597	0.900	0.998	0.999	0.994	0.999	0.836	0.891	0.695	0.933
	Zscales LR	0.896	0.981	0.823	0.982	0.996	0.990	0.994	1.000	0.897	0.839	0.940	0.968
	Rosetta LR	0.878	0.929	0.839	0.978	0.997	0.991	0.998	1.000	0.923	0.886	0.940	0.985
**DRV**	MLP	0.814	0.474	0.750	0.876	0.987	0.726	0.838	0.976	0.850	0.552	0.762	0.895
	CNN	0.986	1.000	0.917	1.000	0.999	0.984	0.991	0.995	0.967	0.840	1.000	0.988
	BRNN	0.857	0.556	0.833	0.891	1.000	0.996	0.994	0.994	0.933	0.760	0.905	0.983
	Triang RF	0.843	0.571	0.333	0.879	1.000	0.999	0.988	0.999	0.883	0.684	0.619	0.946
	Triang KNN	0.871	1.000	0.250	0.884	0.999	0.999	0.974	0.998	0.925	0.875	0.667	0.926
	Zscales LR	0.943	0.786	0.917	0.981	0.995	0.834	0.997	0.999	0.908	0.667	0.952	0.989
	Rosetta LR	0.957	0.750	1.000	0.977	0.998	0.948	0.991	0.999	0.950	0.792	1.000	0.978
**FPV**	MLP	0.781	0.826	0.731	0.903	0.977	0.872	0.956	0.995	0.844	0.769	0.806	0.899
	CNN	0.938	0.940	0.925	0.983	0.999	0.986	0.997	0.999	0.937	0.924	0.925	0.982
	BRNN	0.875	0.881	0.866	0.955	1.000	0.996	1.000	1.000	0.906	0.861	0.925	0.963
	Triang RF	0.875	0.857	0.896	0.966	0.999	0.997	0.996	1.000	0.906	0.843	0.955	0.948
	Triang KNN	0.844	0.930	0.746	0.911	0.998	0.999	0.994	1.000	0.875	0.924	0.806	0.935
	Zscales LR	0.875	0.873	0.881	0.956	0.993	0.924	0.997	1.000	0.906	0.829	0.955	0.983
	Rosetta LR	0.875	0.873	0.881	0.960	0.996	0.962	0.997	1.000	0.906	0.831	0.955	0.973
**IDV**	MLP	0.727	0.709	0.758	0.859	0.980	0.897	0.960	0.990	0.818	0.744	0.833	0.890
	CNN	0.894	0.863	0.934	0.961	0.999	0.986	0.999	0.999	0.909	0.896	0.923	0.971
	BRNN	0.848	0.796	0.923	0.930	0.998	0.994	0.990	0.998	0.894	0.852	0.945	0.965
	Triang RF	0.879	0.826	0.945	0.964	1.000	0.998	0.999	1.000	0.879	0.828	0.934	0.958
	Triang KNN	0.818	0.887	0.747	0.932	0.998	0.999	0.990	0.999	0.864	0.900	0.824	0.943
	Zscales LR	0.879	0.828	0.945	0.959	0.993	0.943	0.996	1.000	0.894	0.824	0.967	0.974
	Rosetta LR	0.864	0.796	0.956	0.935	0.996	0.965	0.996	0.999	0.909	0.852	0.967	0.977
**LPV**	MLP	0.736	0.661	0.831	0.844	0.979	0.869	0.964	0.979	0.836	0.743	0.891	0.920
	CNN	0.914	0.870	0.966	0.943	0.997	0.985	0.989	0.993	0.900	0.831	0.983	0.954
	BRNN	0.893	0.831	0.966	0.948	0.998	0.993	0.989	0.987	0.907	0.845	0.976	0.963
	Triang RF	0.857	0.778	0.949	0.944	0.999	0.998	0.993	1.000	0.857	0.768	0.949	0.911
	Triang KNN	0.879	0.971	0.797	0.850	0.999	0.999	0.993	0.996	0.821	0.930	0.729	0.878
	Zscales LR	0.900	0.824	0.983	0.964	0.994	0.929	0.996	1.000	0.871	0.774	0.966	0.956
	Rosetta LR	0.857	0.765	0.966	0.948	0.995	0.946	0.996	0.997	0.871	0.774	0.966	0.953
**NFV**	MLP	0.747	0.712	0.802	0.852	0.980	0.892	0.967	0.950	0.871	0.843	0.906	0.956
	CNN	0.976	0.990	0.972	0.999	0.999	0.983	0.997	0.999	0.930	0.950	0.921	0.981
	BRNN	0.894	0.881	0.912	0.955	0.999	0.997	0.997	1.000	0.918	0.886	0.960	0.974
	Triang RF	0.906	0.883	0.932	0.983	0.999	0.998	0.996	1.000	0.906	0.870	0.950	0.962
	Triang KNN	0.847	0.969	0.752	0.972	0.998	0.999	0.994	1.000	0.859	0.933	0.792	0.941

Continued on next page

Table S4 (Continued from previous page)

Drug	Method	In-house				Shen (2016)				Steiner (2020)			
		**Acc**	**Prec**	**Rec**	**AUC**	**Acc**	**Prec**	**Rec**	**AUC**	**Acc**	**Prec**	**Rec**	**AUC**
	Zscales LR	0.906	0.881	0.932	0.964	0.993	0.927	0.997	1.000	0.906	0.862	0.960	0.977
	Rosetta LR	0.882	0.857	0.912	0.943	0.996	0.964	0.997	1.000	0.918	0.878	0.970	0.979
**SQV**	MLP	0.812	0.768	0.873	0.892	0.984	0.906	0.969	0.987	0.847	0.791	0.915	0.913
	CNN	0.929	0.906	0.958	0.978	0.999	0.989	0.997	0.998	0.941	0.918	0.972	0.986
	BRNN	0.906	0.873	0.944	0.960	0.999	0.996	0.994	1.000	0.918	0.890	0.958	0.974
	Triang RF	0.882	0.830	0.944	0.959	1.000	0.998	0.997	1.000	0.906	0.862	0.958	0.964
	Triang KNN	0.835	0.952	0.746	0.909	0.999	0.999	0.994	1.000	0.882	0.949	0.831	0.955
	Zscales LR	0.894	0.847	0.958	0.969	0.994	0.932	0.997	1.000	0.906	0.854	0.972	0.984
	Rosetta LR	0.906	0.865	0.958	0.969	0.996	0.958	0.997	1.000	0.929	0.904	0.958	0.986
**TPV**	MLP	0.800	0.647	1.000	0.885	0.976	0.750	0.889	0.975	0.850	0.647	1.000	0.869
	CNN	0.900	0.789	1.000	0.964	0.998	0.944	0.994	0.999	0.900	0.789	1.000	0.988
	BRNN	0.900	0.789	1.000	0.876	0.998	0.985	0.983	0.999	0.888	0.769	1.000	0.944
	Triang RF	0.863	0.706	1.000	0.959	1.000	0.998	0.994	1.000	0.863	0.706	1.000	0.959
	Triang KNN	0.825	1.000	0.688	0.872	0.998	0.999	0.983	0.999	0.838	1.000	0.706	0.989
	Zscales LR	0.900	0.789	1.000	0.972	0.993	0.857	1.000	1.000	0.900	0.789	1.000	0.969
	Rosetta LR	0.875	0.750	1.000	0.967	0.995	0.914	0.994	1.000	0.913	0.818	1.000	0.975

Table S6: **Pairwise model comparisons using DeLong's test**

Drug	Comparison	AUC 1	AUC 2	Diff	p-value	Sig.
ATV	MLP vs CNN	0.847	0.991	0.144	4.57e-05	***
ATV	MLP vs BRNN	0.847	0.983	0.136	1.35e-04	***
ATV	MLP vs ZSCALES	0.847	0.982	0.135	1.05e-04	***
ATV	MLP vs ROSETTA	0.847	0.978	0.131	1.41e-04	***
ATV	MLP vs RF	0.847	0.968	0.121	4.33e-04	***
ATV	CNN vs KNN	0.991	0.900	0.091	0.024	*
ATV	BRNN vs KNN	0.983	0.900	0.083	0.037	*
ATV	KNN vs ZSCALES	0.900	0.982	0.082	0.036	*
ATV	KNN vs ROSETTA	0.900	0.978	0.079	0.042	*
ATV	KNN vs RF	0.900	0.968	0.068	0.063	NS
ATV	MLP vs KNN	0.847	0.900	0.053	0.244	NS
ATV	CNN vs RF	0.991	0.968	0.022	0.084	NS
ATV	BRNN vs RF	0.983	0.968	0.015	0.184	NS
ATV	RF vs ZSCALES	0.968	0.982	0.014	0.240	NS
ATV	CNN vs ROSETTA	0.991	0.978	0.012	0.173	NS
ATV	RF vs ROSETTA	0.968	0.978	0.010	0.430	NS
ATV	CNN vs ZSCALES	0.991	0.982	0.009	0.207	NS
ATV	CNN vs BRNN	0.991	0.983	0.008	0.177	NS
ATV	BRNN vs ROSETTA	0.983	0.978	0.005	0.662	NS
ATV	ZSCALES vs ROSETTA	0.982	0.978	0.004	0.658	NS
ATV	BRNN vs ZSCALES	0.983	0.982	0.001	0.906	NS
DRV	CNN vs ROSETTA	1.000	0.851	0.149	0.003	**
DRV	ZSCALES vs ROSETTA	0.981	0.851	0.131	0.004	**
DRV	MLP vs CNN	0.876	1.000	0.124	0.009	**
DRV	CNN vs RF	1.000	0.879	0.121	0.014	*
DRV	CNN vs KNN	1.000	0.884	0.116	0.293	NS
DRV	CNN vs BRNN	1.000	0.891	0.109	0.049	*
DRV	MLP vs ZSCALES	0.876	0.981	0.105	0.022	*
DRV	RF vs ZSCALES	0.879	0.981	0.103	0.038	*
DRV	KNN vs ZSCALES	0.884	0.981	0.097	0.379	NS
DRV	BRNN vs ZSCALES	0.891	0.981	0.091	0.088	NS
DRV	BRNN vs ROSETTA	0.891	0.851	0.040	0.491	NS
DRV	KNN vs ROSETTA	0.884	0.851	0.034	0.736	NS
DRV	RF vs ROSETTA	0.879	0.851	0.028	0.571	NS
DRV	MLP vs ROSETTA	0.876	0.851	0.026	0.614	NS
DRV	CNN vs ZSCALES	1.000	0.981	0.019	0.148	NS
DRV	MLP vs BRNN	0.876	0.891	0.014	0.826	NS
DRV	BRNN vs RF	0.891	0.879	0.012	0.848	NS
DRV	MLP vs KNN	0.876	0.884	0.008	0.929	NS
DRV	BRNN vs KNN	0.891	0.884	0.006	0.944	NS
DRV	KNN vs RF	0.884	0.879	0.006	0.951	NS
DRV	MLP vs RF	0.876	0.879	0.002	0.957	NS
FPV	MLP vs CNN	0.885	0.997	0.112	1.08e-04	***
FPV	MLP vs BRNN	0.885	0.991	0.106	2.11e-04	***
FPV	MLP vs ZSCALES	0.885	0.989	0.104	4.06e-04	***
FPV	MLP vs ROSETTA	0.885	0.975	0.090	0.003	**
FPV	MLP vs RF	0.885	0.955	0.070	0.018	*
FPV	MLP vs KNN	0.885	0.943	0.059	0.028	*
FPV	CNN vs KNN	0.997	0.943	0.053	0.037	*
FPV	BRNN vs KNN	0.991	0.943	0.047	0.059	NS
FPV	KNN vs ZSCALES	0.943	0.989	0.045	0.070	NS
FPV	CNN vs RF	0.997	0.955	0.042	0.015	*
FPV	BRNN vs RF	0.991	0.955	0.036	0.028	*

Continued on next page

Table S6 – continued from previous page

Drug	Comparison	AUC 1	AUC 2	Diff	p-value	Sig.
FPV	RF vs ZSCALES	0.955	0.989	0.034	0.026	*
FPV	KNN vs ROSETTA	0.943	0.975	0.032	0.203	NS
FPV	CNN vs ROSETTA	0.997	0.975	0.021	0.048	*
FPV	RF vs ROSETTA	0.955	0.975	0.021	0.222	NS
FPV	BRNN vs ROSETTA	0.991	0.975	0.015	0.166	NS
FPV	ZSCALES vs ROSETTA	0.989	0.975	0.013	0.076	NS
FPV	KNN vs RF	0.943	0.955	0.011	0.618	NS
FPV	CNN vs ZSCALES	0.997	0.989	0.008	0.163	NS
FPV	CNN vs BRNN	0.997	0.991	0.006	0.260	NS
FPV	BRNN vs ZSCALES	0.991	0.989	0.002	0.742	NS
IDV	MLP vs CNN	0.894	0.999	0.105	1.13e-05	***
IDV	MLP vs BRNN	0.894	0.987	0.094	8.13e-05	***
IDV	MLP vs ZSCALES	0.894	0.971	0.077	0.001	**
IDV	MLP vs ROSETTA	0.894	0.959	0.066	0.002	**
IDV	MLP vs KNN	0.894	0.956	0.063	0.005	**
IDV	CNN vs RF	0.999	0.945	0.054	0.004	**
IDV	MLP vs RF	0.894	0.945	0.052	0.049	*
IDV	CNN vs KNN	0.999	0.956	0.043	0.037	*
IDV	BRNN vs RF	0.987	0.945	0.042	0.011	*
IDV	CNN vs ROSETTA	0.999	0.959	0.039	0.002	**
IDV	BRNN vs KNN	0.987	0.956	0.031	0.091	NS
IDV	CNN vs ZSCALES	0.999	0.971	0.028	0.011	*
IDV	BRNN vs ROSETTA	0.987	0.959	0.028	0.011	*
IDV	RF vs ZSCALES	0.945	0.971	0.025	0.136	NS
IDV	BRNN vs ZSCALES	0.987	0.971	0.016	0.109	NS
IDV	KNN vs ZSCALES	0.956	0.971	0.015	0.465	NS
IDV	RF vs ROSETTA	0.945	0.959	0.014	0.358	NS
IDV	CNN vs BRNN	0.999	0.987	0.012	0.043	*
IDV	ZSCALES vs ROSETTA	0.971	0.959	0.011	0.068	NS
IDV	KNN vs RF	0.956	0.945	0.011	0.615	NS
IDV	KNN vs ROSETTA	0.956	0.959	0.003	0.866	NS
LPV	MLP vs BRNN	0.911	0.986	0.075	0.009	**
LPV	MLP vs ZSCALES	0.911	0.986	0.075	0.005	**
LPV	MLP vs ROSETTA	0.911	0.975	0.065	0.032	*
LPV	MLP vs CNN	0.911	0.966	0.056	0.097	NS
LPV	MLP vs KNN	0.911	0.962	0.051	0.095	NS
LPV	MLP vs RF	0.911	0.960	0.049	0.087	NS
LPV	BRNN vs RF	0.986	0.960	0.026	0.089	NS
LPV	RF vs ZSCALES	0.960	0.986	0.026	0.132	NS
LPV	BRNN vs KNN	0.986	0.962	0.024	0.144	NS
LPV	KNN vs ZSCALES	0.962	0.986	0.024	0.168	NS
LPV	CNN vs BRNN	0.966	0.986	0.020	0.211	NS
LPV	CNN vs ZSCALES	0.966	0.986	0.019	0.307	NS
LPV	RF vs ROSETTA	0.960	0.975	0.015	0.370	NS
LPV	KNN vs ROSETTA	0.962	0.975	0.014	0.434	NS
LPV	BRNN vs ROSETTA	0.986	0.975	0.011	0.195	NS
LPV	ZSCALES vs ROSETTA	0.986	0.975	0.010	0.327	NS
LPV	CNN vs ROSETTA	0.966	0.975	0.009	0.576	NS
LPV	CNN vs RF	0.966	0.960	0.006	0.747	NS
LPV	CNN vs KNN	0.966	0.962	0.005	0.753	NS
LPV	KNN vs RF	0.962	0.960	0.002	0.909	NS
LPV	BRNN vs ZSCALES	0.986	0.986	0.000	0.989	NS
NFV	MLP vs CNN	0.888	0.999	0.112	6.10e-06	***
NFV	MLP vs BRNN	0.888	0.996	0.108	1.02e-05	***

Continued on next page

Table S6 – continued from previous page

Drug	Comparison	AUC 1	AUC 2	Diff	p-value	Sig.
NFV	MLP vs ZSCALES	0.888	0.990	0.102	1.51e-05	***
NFV	MLP vs ROSETTA	0.888	0.981	0.093	9.89e-05	***
NFV	MLP vs RF	0.888	0.979	0.091	6.26e-04	***
NFV	CNN vs KNN	0.999	0.939	0.060	0.033	*
NFV	BRNN vs KNN	0.996	0.939	0.056	0.045	*
NFV	MLP vs KNN	0.888	0.939	0.051	0.117	NS
NFV	KNN vs ZSCALES	0.939	0.990	0.051	0.055	NS
NFV	KNN vs ROSETTA	0.939	0.981	0.042	0.124	NS
NFV	KNN vs RF	0.939	0.979	0.040	0.046	*
NFV	CNN vs RF	0.999	0.979	0.020	0.252	NS
NFV	CNN vs ROSETTA	0.999	0.981	0.018	0.013	*
NFV	BRNN vs RF	0.996	0.979	0.017	0.351	NS
NFV	BRNN vs ROSETTA	0.996	0.981	0.015	0.051	NS
NFV	RF vs ZSCALES	0.979	0.990	0.011	0.519	NS
NFV	CNN vs ZSCALES	0.999	0.990	0.010	0.050	NS
NFV	ZSCALES vs ROSETTA	0.990	0.981	0.009	0.197	NS
NFV	BRNN vs ZSCALES	0.996	0.990	0.006	0.166	NS
NFV	CNN vs BRNN	0.999	0.996	0.004	0.176	NS
NFV	RF vs ROSETTA	0.979	0.981	0.002	0.902	NS
SQV	MLP vs CNN	0.916	0.998	0.082	6.62e-05	***
SQV	MLP vs BRNN	0.916	0.990	0.074	2.52e-04	***
SQV	MLP vs ROSETTA	0.916	0.985	0.069	9.47e-04	***
SQV	MLP vs ZSCALES	0.916	0.983	0.067	6.79e-04	***
SQV	MLP vs RF	0.916	0.967	0.051	0.016	*
SQV	CNN vs KNN	0.998	0.955	0.044	0.029	*
SQV	MLP vs KNN	0.916	0.955	0.039	0.061	NS
SQV	BRNN vs KNN	0.990	0.955	0.035	0.060	NS
SQV	CNN vs RF	0.998	0.967	0.031	0.045	*
SQV	KNN vs ROSETTA	0.955	0.985	0.030	0.098	NS
SQV	KNN vs ZSCALES	0.955	0.983	0.028	0.119	NS
SQV	BRNN vs RF	0.990	0.967	0.023	0.105	NS
SQV	RF vs ROSETTA	0.967	0.985	0.018	0.168	NS
SQV	RF vs ZSCALES	0.967	0.983	0.015	0.239	NS
SQV	CNN vs ZSCALES	0.998	0.983	0.015	0.071	NS
SQV	CNN vs ROSETTA	0.998	0.985	0.013	0.084	NS
SQV	KNN vs RF	0.955	0.967	0.013	0.422	NS
SQV	CNN vs BRNN	0.998	0.990	0.008	0.092	NS
SQV	BRNN vs ZSCALES	0.990	0.983	0.007	0.318	NS
SQV	BRNN vs ROSETTA	0.990	0.985	0.005	0.458	NS
SQV	ZSCALES vs ROSETTA	0.983	0.985	0.002	0.610	NS
TPV	CNN vs KNN	0.988	0.832	0.156	0.051	NS
TPV	CNN vs ROSETTA	0.988	0.844	0.144	0.021	*
TPV	MLP vs CNN	0.857	0.988	0.131	0.003	**
TPV	CNN vs RF	0.988	0.866	0.122	0.009	**
TPV	BRNN vs KNN	0.934	0.832	0.102	0.154	NS
TPV	BRNN vs ROSETTA	0.934	0.844	0.090	0.065	NS
TPV	CNN vs ZSCALES	0.988	0.901	0.087	0.076	NS
TPV	MLP vs BRNN	0.857	0.934	0.077	0.037	*
TPV	KNN vs ZSCALES	0.832	0.901	0.069	0.307	NS
TPV	BRNN vs RF	0.934	0.866	0.068	0.042	*
TPV	ZSCALES vs ROSETTA	0.901	0.844	0.057	0.188	NS
TPV	CNN vs BRNN	0.988	0.934	0.054	0.039	*
TPV	MLP vs ZSCALES	0.857	0.901	0.043	0.379	NS
TPV	RF vs ZSCALES	0.866	0.901	0.034	0.457	NS

Continued on next page

Table S6 – continued from previous page

Drug	Comparison	AUC 1	AUC 2	Diff	p-value	Sig.
TPV	KNN vs RF	0.832	0.866	0.034	0.576	NS
TPV	BRNN vs ZSCALES	0.934	0.901	0.033	0.447	NS
TPV	MLP vs KNN	0.857	0.832	0.025	0.719	NS
TPV	RF vs ROSETTA	0.866	0.844	0.022	0.687	NS
TPV	MLP vs ROSETTA	0.857	0.844	0.013	0.798	NS
TPV	KNN vs ROSETTA	0.832	0.844	0.012	0.881	NS
TPV	MLP vs RF	0.857	0.866	0.009	0.838	NS

Table S7: **Model coefficients for NFV Rosetta logistic regression model trained using the In-house dataset**

**Feature**	**Coefficient**	**p-value**
24-ScoreType.fa_dun	22.08	0.021
52-ScoreType.rama_prepro	16.83	0.094
45-ScoreType.fa_dun	12.00	0.006
84-ScoreType.fa_atr	11.48	3.63×10^{-6}
54-ScoreType.fa_dun	10.49	0.039
46-ScoreType.cart_bonded	-10.15	0.003
54-ScoreType.lk_ball_wtd	9.99	0.036
52-ScoreType.fa_atr	-8.50	0.328
53-ScoreType.lk_ball_wtd	-6.48	0.260
10-ScoreType.fa_intra_sol_xover4	6.02	0.047
54-ScoreType.rama_prepro	-5.58	0.127
45-ScoreType.p_aa_pp	5.35	0.301
47-ScoreType.omega	5.24	0.430
55-ScoreType.omega	-4.68	0.195
53-ScoreType.fa_dun	-4.48	0.431
54-ScoreType.fa_atr	-3.93	0.494
48-ScoreType.fa_elec	3.90	0.403
53-ScoreType.p_aa_pp	-3.59	0.600
21-ScoreType.fa_atr	-3.42	0.009
55-ScoreType.fa_atr	3.27	0.475
82-ScoreType.fa_intra_rep	3.11	0.086
48-ScoreType.fa_sol	-2.98	0.714
90-ScoreType.fa_intra_rep	-2.89	0.159
47-ScoreType.fa_intra_sol_xover4	2.60	0.626
32-ScoreType.fa_sol	2.52	0.506
53-ScoreType.fa_sol	-2.13	0.797
53-ScoreType.omega	-2.00	0.740
9-ScoreType.fa_elec	-1.82	0.438
45-ScoreType.rama_prepro	1.48	0.721
24-ScoreType.fa_sol	1.39	0.561
81-ScoreType.fa_intra_sol_xover4	-1.32	0.597
53-ScoreType.fa_rep	-0.79	0.889
46-ScoreType.p_aa_pp	-0.32	0.921
10-ScoreType.p_aa_pp	-0.24	0.872
78-ScoreType.fa_rep	-0.18	0.945
84-ScoreType.fa_dun	0.13	0.933
45-ScoreType.fa_elec	0.10	0.972

Table S8: **Model coefficients for NFV zScales logistic regression model trained using the In-house dataset**

Feature	Formated Feature	Coefficient	p-value
V150	30_5	5.12	0.886
V352	71_2	-1.89	0.971
V238	48_3	-1.43	0.073
V50	10_5	-1.38	0.171
V416	84_1	1.35	0.937
V366	74_1	1.27	0.222
V439	88_4	-1.22	0.823
V266	54_1	1.05	0.957
V365	73_5	0.92	0.996
V408	82_3	0.88	0.842
V269	54_4	0.86	0.978
V449	90_4	0.84	0.973
V226	46_1	-0.75	0.981
V367	74_2	0.73	0.582
V437	88_2	-0.63	0.864
V407	82_2	0.55	0.715
V176	36_1	-0.53	0.868
V304	61_4	-0.50	0.285
V247	50_2	-0.49	0.654
V362	73_2	0.47	0.999
V98	20_3	-0.40	0.946
V355	71_5	-0.38	0.975
V229	46_4	-0.38	0.985
V211	43_1	0.31	0.582
V392	79_2	0.25	0.890
V315	63_5	0.20	0.639
V202	41_2	0.18	0.566
V58	12_3	-0.17	0.534
V120	24_5	-0.17	0.995
V96	20_1	-0.15	0.685
V174	35_4	0.14	0.911
V313	63_3	0.12	0.593
V162	33_2	-0.11	0.995
V282	57_2	0.07	0.983
V47	10_2	0.06	0.947
V99	20_4	-0.06	0.992
V406	82_1	-0.06	0.971
V182	37_2	0.06	0.766
V175	35_5	0.05	0.958

2 Supplementary Figures

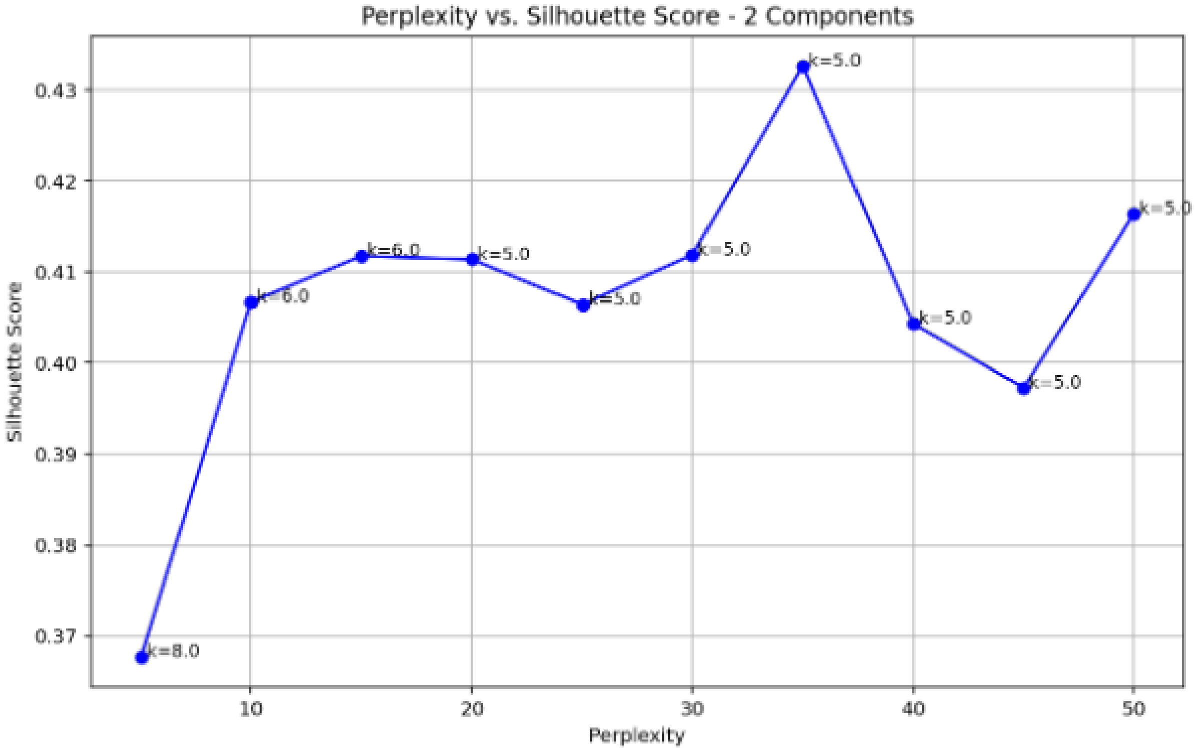

Figure S1: **Optimization of t-SNE parameters for cluster analysis of HIV-1 protease sequences**. The plot shows silhouette scores (y-axis) for different perplexity values (x-axis) in t-SNE dimensionality reduction to 2 components. Each point represents a combination of perplexity and optimal k value for K-means clustering, with the k value annotated next to each point. The highest silhouette score (0.433) was achieved with perplexity=35 and k=5, indicating optimal cluster separation and cohesion. This parameter combination was selected for the clustering-based validation strategy to ensure comprehensive representation of sequence diversity in the test set construction.
